# MicroRNA-204-5p reduction in rat hippocampus contributes to stress-induced pathology via targeting RGS12 signaling pathway

**DOI:** 10.1186/s12974-021-02299-5

**Published:** 2021-10-21

**Authors:** Tian Lan, Ye Li, Cuiqin Fan, Liyan Wang, Wenjing Wang, Shihong Chen, Shu Yan Yu

**Affiliations:** 1 Department of Physiology, School of Basic Medical Sciences, Shandong University, 44 Wenhuaxilu Road, Jinan, Shandong 250012 People’s Republic of China; 2 Morphological Experimental Center, School of Basic Medical Sciences, Shandong University, 44 Wenhuaxilu Road, Jinan, Shandong 250012 People’s Republic of China; 3 Department of Endocrinology, The Second Hospital, Cheeloo College of Medicine, Shandong University, 247 Beiyuan Street, Jinan, Shandong 250033 People’s Republic of China; 4 Shandong Provincial Key Laboratory of Mental Disorders, School of Basic Medical Sciences, 44 Wenhuaxilu Road, Jinan, Shandong 250012 People’s Republic of China

**Keywords:** MiR-204-5p, Neuroinflammation, Oxidative stress, RGS12, Depression

## Abstract

**Background:**

Neuroinflammation occupies a pivotal position in the pathogenesis of most nervous system diseases, including depression. However, the underlying molecular mechanisms of neuroinflammation associated with neuronal injury in depression remain largely uncharacterized. Therefore, identifying potential molecular mechanisms and therapeutic targets would serve to better understand the progression of this condition.

**Methods:**

Chronic unpredictable stress (CUS) was used to induce depression-like behaviors in rats. RNA-sequencing was used to detect the differentially expressed microRNAs. Stereotactic injection of AAV virus to overexpress or knockdown the miR-204-5p. The oxidative markers and inflammatory related proteins were verified by immunoblotting or immunofluorescence assay. The oxidative stress enzyme and products were verified using enzyme-linked assay kit. Electron microscopy analysis was used to observe the synapse and ultrastructural pathology. Finally, electrophysiological recording was used to analyze the synaptic transmission.

**Results:**

Here, we found that the expression of miR-204-5p within the hippocampal dentate gyrus (DG) region of rats was significantly down-regulated after chronic unpredicted stress (CUS), accompanied with the oxidative stress-induced neuronal damage within DG region of these rats. In contrast, overexpression of miR-204-5p within the DG region of CUS rats alleviated oxidative stress and neuroinflammation by directly targeting the regulator of G protein signaling 12 (RGS12), effects which were accompanied with amelioration of depressive-like behaviors in these CUS rats. In addition, down-regulation of miR-204-5p induced neuronal deterioration in DG regions and depressive-like behaviors in rats.

**Conclusion:**

Taken together, these results suggest that miR-204-5p plays a key role in regulating oxidative stress damage in CUS-induced pathological processes of depression. Such findings provide evidence of the involvement of miR-204-5p in mechanisms underlying oxidative stress associated with depressive phenotype.

**Graphical Abstract:**

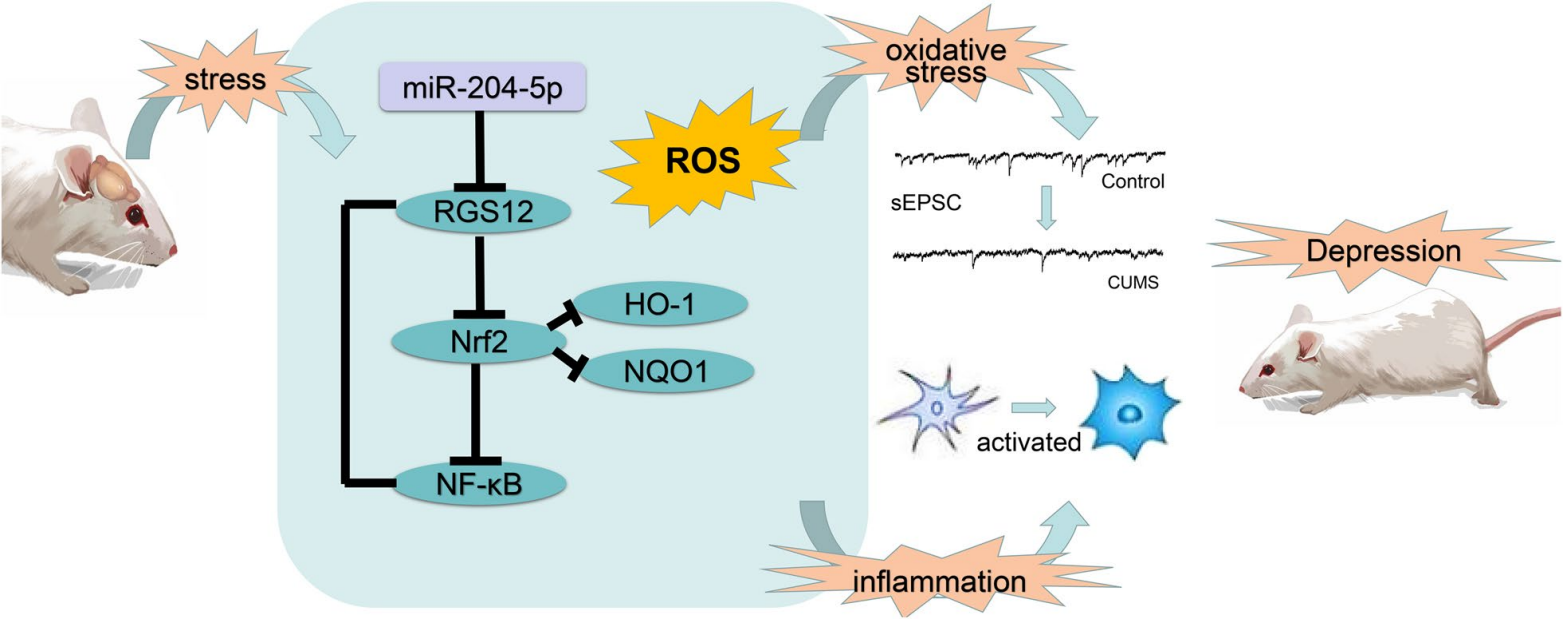

**Supplementary Information:**

The online version contains supplementary material available at 10.1186/s12974-021-02299-5.

## Background

Depression is a major pervasive mental disorder mainly manifested as a persistent despondent mood and brain-related dysfunction [1]. Identifying the exact etiology of major depressive disorder (MDD) is a challenge, with results from previous studies indicating that the pathophysiological processes of depression are associated with neuronal damage caused by inflammation and oxidative stress, neurotransmitter changes and synaptic plasticity abnormalities [2–7].

The brain with its rich lipid content, high energy requirements and low antioxidant capacity is a particularly vulnerable site for excessive oxidative damage [8]. Results from clinical studies have provided evidence of oxidative stress damage in the brain and peripheral blood of patients with depression [9, 10]. This link between oxidative stress and depression represents a significant issue underlying a better understanding of the pathophysiology of depression.

MicroRNAs (miRNAs) are small non-coding RNA molecules that regulate the activity of approximately half of all protein-coding genes and thus regulate fluctuations in protein expression [11, 12]. Changes in expression levels of specific miRNAs have been observed as being associated with the development of various central nervous system (CNS) diseases [13]. Of particular relevance to the present report are the clinical findings of alterations in many miRNAs within the brains of patients with depression [14]. By regulating relative changes in miRNA levels involved with pathological processes, there is a possibility to reduce pathological damage and thus achieve a level of neuroprotection [15]. In this way, the elucidation of interactive mechanisms among oxidative stress, miRNAs and their targeted genes, as associated with specific neurological diseases, would be of great significance for a targeted treatment of these disorders.

In this study, with use of high-throughput sequencing and Kyoto Encyclopedia of Genes and Genomes (KEEG) database analysis, differentially expressed miRNAs as obtained in a rat model of depression versus normal rats were identified. We found that one specific miRNA, miR-204-5p, which may serve as one of the factors that affect the occurrence of depression by regulating oxidative stress in the brain, was significantly reduced within the hippocampal DG region in the chronic unpredicted stress (CUS) induced rat model of depression. Interestingly, a down-regulation of miR-204-5p induced increased expression of its downstream target gene, RGS12, and caused the incidence of oxidative damage and inflammation within the hippocampal DG region possibly via regulating the Nrf2/NF-κB signaling pathway. Conversely, an up-regulation of miR-204-5p attenuates depression-like behaviors, reduces oxidative damage and neuroinflammatory responses within the DG region, as well as augmenting synaptic plasticity in this rat model of depression.

## Methods

### Animals and housing conditions

Male Wistar rats weighing 120–140 g (about 5 weeks) were purchased from the Experimental Animal Centre of Shandong University. All experiments were approved by the Shandong University Animal Care and Use Committee and were conducted according to the International Guiding Principles for Animal Research provided by the International Organizations of the Medical Sciences Council. Rats were housed under a 12 h light/dark cycle and had free access to food and water. Ambient temperature was maintained at 22 ℃ ± 2 ℃, except when subjected to the conditions of specific experiments. All efforts were made to minimize the pain and numbers of the animals used in the experiments.

### CUS model

Rats in the CUS group were subjected to procedures described previously [16]. Briefly, these rats were individually housed and subjected to a daily stress regime for 5 weeks consisting of: (1) 24 h of water and food deprivation, (2) swimming in cold (4 °C) water for 5 min, (3) damp sawdust for 24 h, (4) tail clamp for 3 min, (5) cage tilted 45°for 12 h, (6) restricted movement for 2 h and (7) horizontal oscillation for 5 min. One stressor was applied per day in a random order. Rats in the control group were housed under laboratory conditions.

### Behavioral tests

All behavioral experiments were conducted in isolated behavioral testing rooms and performed by experimenters who were blind as to the identity of the experimental groups.

#### Forced swimming test

The forced swimming test (FST) was used to evaluate depression-like behaviors. After the 5-week period of stress administration, rats were placed individually in a water filled cylinder (height: 80 cm, diameter: 30 cm, 25 °C) for 15 min of forced swimming in the training session. At 24 h after this training session, the rats were again placed in the water filled cylinder for the 5 min test session. During this 5 min test session, immobility and horizontal movement times were recorded. Rats floating motionless without struggling or making minimal movements only necessary to maintain their heads above the water surface were considered to be immobile. Horizontal movement throughout the cylinder was defined as swimming while vertical movement against the wall of the cylinder was defined as climbing.

#### Sucrose preference test

The sucrose preference test (SPT) was performed 5 weeks after CUS treatment as a means of assessing anhedonia symptoms in rats. As described in the previous literature with minor modifications [17]. Briefly, rats were placed individually in cages with two bottles of sucrose solution (1%, w/v) for the first 24 h period and then one bottle of sucrose solution was replaced with tap water for the second 24 h period. After this adaptation phase, rats were deprived of food and water for 24 h and then permitted access to two bottles for 3 h, one bottle containing 100 ml of 1% sucrose solution and the other containing 100 ml of tap water. Sucrose preference was defined as sucrose consumption/[water consumption + sucrose consumption] × 100% during the test phase.

#### Morris water maze

The Morris water maze (MWM) test was performed to evaluate spatial memory and learning ability in an aqueous environment. The water maze, a cylindrical pool (60 cm in radius, 50 cm in height), was visually separated into four quadrants. A platform with a diameter of 13 cm was placed 1 cm below the waterline in the center of one quadrant below the water surface. During the 5 days of training sessions, each rat was placed at one of four start locations facing the wall of the pool and trained to locate the platform in the fourth quadrant within 60 s and then remained on the platform for 30 s. If the rat failed to locate the platform within 60 s, it was guided to the platform and was required to remain there for 15 s. The sixth day was the test day. The platform was withdrawn, and the rat was placed in the opposite quadrant of the goal quadrant to swim freely for 60 s. Defines the platform as the target quadrant, and SMART records the time within the target quadrant and the number of times across the platform (SMART is a video tracking platform software).

### Dual-luciferase reporter assay

The experimental plasmid was constructed by RiboBio Technology (Guangzhou). Luciferase reporter assays were performed according to the following description. First, the 3′-UTR sequences of wild-type (WT) or mutant-type (MUT) RGS12s were cloned into the luciferase reporter plasmid, and were co-transfected with the miR-204-5p mimic or miR-NC into HEK-293 T cells for 48 h. To analyze interactions between miR-204-5p and RGS12, firefly luciferase activities were measured after transfection using the Dual-Luciferase Reporter Assay System. All luciferase values were normalized to those of firefly luciferase and expressed as fold induction relative to basal activity.

### miRNA library construction and sequencing

According to the manufacturer's instructions, total RNA was isolated from hippocampal DG region tissues using Trizol kit (Invitrogen) for the preparation and sequencing of miRNA libraries. The RNA integrity (RIN) was accessed using Agilent 2200 Tapestation (Agilent Technologies, USA). Small RNAs were reverse transcribed into cRNAs and amplified by PCR. The PCR products were sequenced using the HiSeq 2500 and HiSeq 3000 (Illumina, USA) platform at the RiboBio Company (Guangzhou, China). The up-regulated and down-regulated MicroRNAs with significant differences in expression ranked by fold changes in microarray are listed in Additional file 2: Table S2.

Differentially expressed microRNAs in the sequencing results were analyzed by Diana miRpath. MicroRNAs and pathways were classified according to their interaction levels, and these results were combined by combination and meta-analysis algorithms[18]. Used three different databases, miRTARBASE, miRDB and TargetScan, to predict the possible target genes downstream of miR-204-5p, screened the same target genes predicted in the three databases. MiRNA target genes implicated in the pathway were investigated among genes with use of the KEGG database [19]. A *P* < 0.05 was used as the criterion for statistical significance.

### Stereotactic injection of the AAV virus

After completion of behavioral tests, rats in the CUS and control groups were randomly selected for virus injection. Different groups of rats were randomly selected by non-participants. The AAV9-virus was obtained from GENEchem (Shanghai, China). The AAV9-rno-miR-204-5p virus (primer sequence: UUCCCUUUGUCAUCCUAUGCCU) was constructed to overexpress miR-204-5p while the AAV9-rno-miR-204-5p-sponge virus (inverse complementary sequence: AGGCATAGGATGACAAAGGGAA) was used to block miR-204-5p in the DG region of the hippocampus. Rats were anesthetized with an intraperitoneal injection of 2.5% isoflurane as based on their body weights and then positioned within the stereotaxic apparatus (Stoelting, USA). Small burr holes were drilled on two sides of the skull (3.24 mm posterior to bregma and 1.8 mm lateral to the midline) to allow access to the hippocampal DG region for injection of the AAV virus (~ 10^12^ infection units per ml, a flow rate of 140 nl/min) at the depth of 3.5 mm. Behavioral tests or biochemical assays were performed at ≥ 14 days after injection. The injection sites were verified after behavioral tests and only rats with correct injection sites were used for analyses in the subsequent assays.

### Brain anatomy and tissue preparation

Twenty-four hours after behavioral tests, rats from each group were anesthetized with sodium phenobarbital (30 mg/kg) and transcardially perfused with 300 ml 0.9% NaCl containing heparin sodium salt followed by fixation with 4% paraformaldehyde (PFA). Brains were removed and post-fixed in 4% PFA overnight at 4℃followed by a graded dehydration. Brain tissue was encased in optimum cutting temperature compound and frozen serial coronal sections were cut. Sections, consisting of prefrontal cortex and hippocampus, were selected for immunofluorescent staining.

### Immunofluorescent staining and confocal microscopy

Frozen slices (40 μm) were incubated overnight at 4 ℃ with the following antibodies: rabbit anti-NeuN (24,307, Cell Signaling Technology), mouse anti-NeuN (ab104224, Abcam), rabbit anti-ionized calcium binding adaptor molecule-1 (Iba-1, 019-19741, Wako Pure Chemical Inc), mouse anti-RGS12 (sc-398545, Santa Cruz Biotechnology) or rabbit anti-MAP2 (4542, Cell Signaling Technology). Sections were then incubated with Alexa Fluor 488 or 546 conjugated (Abcam) or alexa-568 (Invitrogen), for 1 h at 37℃ in a thermostatic oscillator. DAPI (Beyotime) was used for nuclear staining. Before each step, slices were washed three times in PBS. Images were captured with use of a confocal microscope (LSM880, Carl Zeiss, Germany) and processed using ZEN software. The fluorescent intensity analysis was performed using ImageJ (NIH, Bethesda, MD, USA). The positive cells of Iba1 per 1 mm^2^ were counted by experimenters used ImageJ, blind to the treatment conditions. All experiments were conducted in a blinded manner, adhering to stereological principles.

### Western blotting

Rats were anesthetized with an intraperitoneal injection of sodium phenobarbital (30 mg/kg) as based on their body weight. Inside the hippocampus, CA1 region is located on the dorsal side of the brain and DG is located on the ventral side of the brain. Under the stereomicroscope, CA1 and DG region could be divided along the hippocampal fissure between them in the ventral surface of the hippocampus. Bilateral DG tissue samples were isolated and lysed in RIPA buffer containing a cocktail of protease/phosphatase inhibitors. After centrifugation (20 min, 12,000 rpm, 4 ℃), cleared lysates containing the isolated proteins were harvested. Protein concentrations were determined using the Pierce BCA protein kit. Western blot was performed as described previously [20]. Proteins (30 μg) from each tissue sample were loaded in each lane, electrophoretically separated on SDS-PAGE gels and then transferred onto PVDF membranes for primary antibody incubation at 4 °C for overnight. Membranes were incubated with the following antibodies: rabbit anti-β-actin (4970, Cell Signaling Technology), rabbit anti-NOX1 (DF8684, Affinity Biosciences), rabbit anti-NOX4 (bs60435, Bioworld), rabbit anti-Nrf2 (ab137550, Abcam), rabbit anti-HO-1 (ab13243, Abcam), mouse anti-RGS12 (sc-398545, Santa Cruz Biotechnology), rabbit anti-NF-κB p65 (bs90940, Bioworld) or mouse anti-NQO1 (ab2894, Abcam). Horseradish peroxidase-conjugated antibodies (Santa Cruz Biotechnology) were used as labelled secondary antibodies. Image-J software was used to perform pixel quantification of the images. Intra-run normalization against the internal actin control was performed for each sample.

### Real-time quantitative PCR

Expression levels of miRNA were determined using quantitative real-time quantitative PCR with The All-in-OneTM miRNA qRT-PCR Detection Kit (GeneCopoeia, USA) and mRNA qRT-PCR detection kit (GeneCopoeia, USA) was used to determine mRNA expression level in real-time quantitative PCR. Real-time quantitative PCR analysis was performed on a Bio-Rad iCycler system (Bio-Rad, Hercules, CA). Rno-U6 served as a loading control for the sample to test for miRNA and GAPDH served as a loading control for the sample to test for mRNA, mRNA expression levels and miRNA expression levels were evaluated using the 2 − (ΔΔCt) method. Sequences of specific primers are listed in Additional file 1: Table S1.

### Transmission electron microscopy (TEM)

The ultrastructure of DG neurons was observed with use of transmission electron microscopy (Philips Tecnai 20 U-Twin, Holland). Bilateral DG tissues were carefully dissected (1 × 1 × 1 mm), fixed with 1% osmium tetroxide for 2 h, and dehydrated with graded ethanol. The tissue was infiltrated overnight with a semi-epoxy-propane mixture and then embedded in resin. Tissues were cut into ultrathin sections (70 nm), stained with 4% uranyl acetate for 20 min and then stained with 0.5% lead citrate on the copper grid. At least 30 micrographs were randomly selected from each rat and analyzed using Image J analysis software (NIH, Scion Corporation, Frederick, MD).

### Acute hippocampal slice preparation and electrophysiological analysis

Rats were anesthetized using 2.5% isoflurane and decapitated. The brain was extracted, blocked, placed on a vibrating slicer, and immersed in a cold solution containing (in mM) 119 choline chloride, 30 Glucose, 26 NaHCO_3_, 7 MgSO_4_, 2.5 KCl, 1 NaH_2_PO_4_, 1 CaCl_2_, 3 sodiumpyruvate, 1.3 sodium L-ascorbate, 1 kynurenicacid, and saturated with 95% O2/5% CO_2_. Slices were then transferred as quickly as possible to a recovery solution containing (in mM) 85 NaCl, 24 NaHCO_3_, 4 MgCl_2_, 2.5 KCl, 1.25 NaH_2_PO_4_, 0.5 CaCl_2_, 25 glucose and 50 sucrose and allowed to recover for 30 min at 30 °C. The glass micropipettes (4–6 MΩ) were filled with an internal solution containing (in mM) 130 CsMeSO_4_, 10 CsCl, 4 NaCl, 1 MgCl_2_, 5 MgATP, 5 EGTA, 10 HEPES, 0.5 Na3GTP, 10 phosphocreatine and 4 QX-314, with a pH of 7.35. During whole-cell clamp patch recordings, slices were continuously perfused with an artificial cerebral spinal fluid (ACSF) contained (in mM) 120 NaCl, 3.5 KCl, 2.5 CaCl_2_, 1.3 MgSO_4_, 1.25 NaH_2_PO_4_, 26 NaHCO3 and 10 glucose.

### Oxidative stress measures

#### ROS measurement

To measure the ROS production in tissue, frozen slices were incubated with 10 μM dihydroethidium (DHE) for 30 min at 37 ℃ and then stained with DAPI (Beyotime) for 10 min. Mitochondrial ROS levels were measured with use of 10 μM MitoSOX Red fluorescent dye for 15 min at 37 ℃ in a thermostatic oscillator, followed by staining with DAPI (ThermoFisher) for 10 min. Images were captured on a Zeiss LSM 880 scanning laser confocal microscope.

#### Antioxidant enzyme activities

Activity of antioxidant enzymes in CA1, DG and vmPFC tissue was measured using the superoxide dismutase (SOD, No. A001-3), lactic dehydrogenase (LDH, No. A020-2) and total antioxidant capacity (T-AOC, A015-2) activity assay kits according to the manufacturers’ guidelines. All assay kits were purchased from Jiancheng Inc. (Nanjing, China).

#### Assessment of DNA damage markers

Oxidative DNA damage in DG regions was then detected using an immunofluorescent assay. Frozen slices were incubated with the primary mouse anti-DNA/RNA Damage antibody (ab62623 Abcam) and rabbit anti-NeuN overnight at 4 ℃ followed by Alexa Fluor 488 or 546 conjugated (Abcam). DAPI (Beyotime) was used for nuclear staining.

#### Oxidative stress products

The lipid peroxidation product resulting from oxidative stress, 4-HNE, was measured using immunofluorescent staining with polyclonal goat anti-4-hidroxynonenal (4-HNE, ab46545, Abcam). In addition, CA1, DG and mPFC tissues were homogenized for determination of MDA content (No. A003-1) as performed using assay kits from Jiancheng Inc. (Nanjing, China). All samples were assayed in duplicate and results were normalized to total protein content.

### Statistical analysis

All calculations were performed using GraphPad Prism 8.0.1 (GraphPad Software, Inc., San Diego, CA). All data were presented as the means ± standard errors of the mean. Statistical significance of differences in the groups was evaluated with use of either the Student's *t* test, two-way analysis of variance (ANOVA) or one-way analysis of variance (ANOVA) with the Tukey's multiple-comparison test used for post-hoc comparisons. A value of *P* < 0.05 was required for results to be considered statistically significant.

## Results

### Oxidative damage was enhanced in specific brain regions of CUS rats

**Fig. 1:**
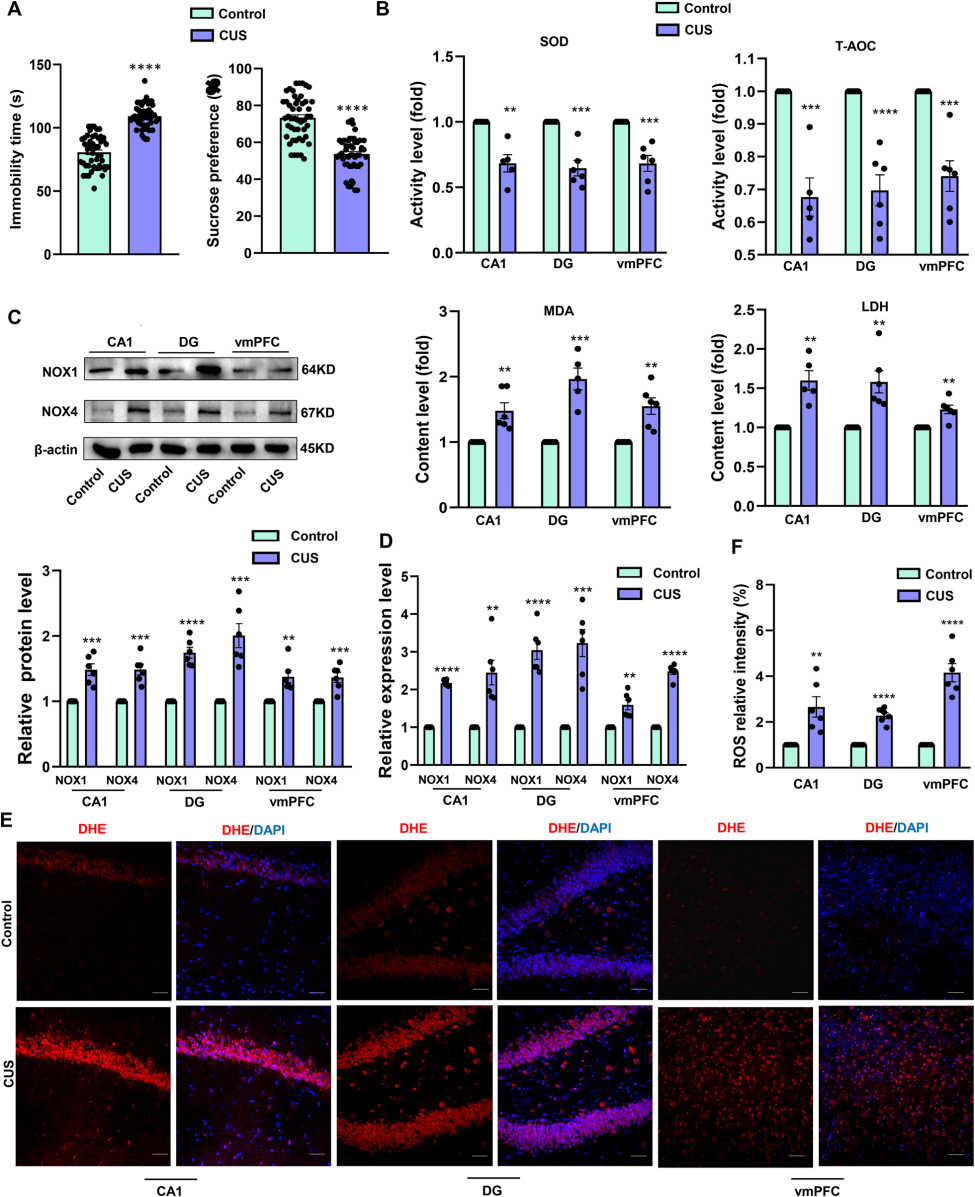
Oxidative damage as observed in the hippocampal DG region and vmPFC of CUS rats. **A** Behavioral responses of FST and SPT in control rats and CUS model rats (*N* = 50 per group). **B** Activity of antioxidant enzymes SOD and T-AOC. Contents of MDA and LDH were analyzed and levels were normalized to total protein content (*N* = 5–6 per group). **C** Representative western blot images showing relative protein levels NOX1 and NOX4 (*N* = 6 per group). **D** Q-PCR analysis of NOX1 and NOX4 mRNA levels of each group. Band intensities were normalized to GAPDH (*N* = 6 per group). **E** Representative images of DHE staining (red) within the DG area from each group of rats following ROS analysis (*N* = 6 per group). Nuclei (blue) are stained with DAPI. Scale bar is 50 μm. **F** ROS relative intensity analysis after DHE staining in each group *P < 0.05, **P < 0.01, ***P < 0.001, ****P < 0.0001, vs Control group. Data are presented as means ± SEMs. Student *t*-tests were employed for comparisons between the two groups

Depression is usually accompanied by neuronal damage to specific brain regions and the production of excessive ROS, which can then lead to inflammation and cell apoptosis. After 4–6 weeks of CUS stimulation, the immobility time of CUS rats significantly increased in the forced swimming test (*P* < 0.0001), and the sucrose preference test also confirmed that the CUS rats were experiencing anhedonia (*P* < 0.0001) which indicated depression-like behavior of rat (Fig. 1A). When the activities of a number of antioxidant enzymes in the ventromedial prefrontal cortex (vmPFC), hippocampal CA1 and DG regions were examined in CUS and non-stressed control rats, we found that superoxide dismutase (SOD) (CA1: *P* < 0.01, DG: *P* < 0.001, vmPFC: *P* < 0.001) and total antioxidant capacity (T-AOC) activity (CA1: *P* < 0.001; DG: *P* < 0.0001; vmPFC: *P* < 0.001) were significantly reduced in CUS rats. In addition, increased levels of the oxidative stress products, malondialdehyde (MDA) (CA1: *P* < 0.001; DG: *P* < 0.0001; vmPFC: *P* < 0.001) and lactate dehydrogenase (LDH) (CA1: *P* < 0.001; DG: *P* < 0.0001; vmPFC: *P* < 0.001) were observed within these three regions of CUS rats (Fig. 1B). As NADPH oxidases (NOX) are a major source of ROS, we next examined the expressions of NOX family members and found that the levels of NOX1 (CA1: *P* < 0.0001; DG: *P* < 0.0001; vmPFC: *P* < 0.01) and NOX4 (CA1: *P* < 0.01; DG: *P* < 0.001; vmPFC: *P* < 0.0001) transcripts were significantly increased in these CUS (Fig. 1D). The protein expressions of NOX1 (CA1: *P* < 0.001; DG: *P* < 0.0001; vmPFC: *P* < 0.01) and NOX4 (CA1: *P* < 0.001; DG: *P* < 0.001; vmPFC: *P* < 0.001) were also increased in these CUS (Fig. 1C). Results from immunofluorescent assays also showed the ROS levels within DG regions of CUS rats were increased over that observed in unstressed rats (CA1: *P* < 0.01; DG: *P* < 0.0001; vmPFC: *P* < 0.0001, Fig. 1E, F).

### MiR-204-5p is reduced within the hippocampal DG region of CUS rats

**Fig. 2:**
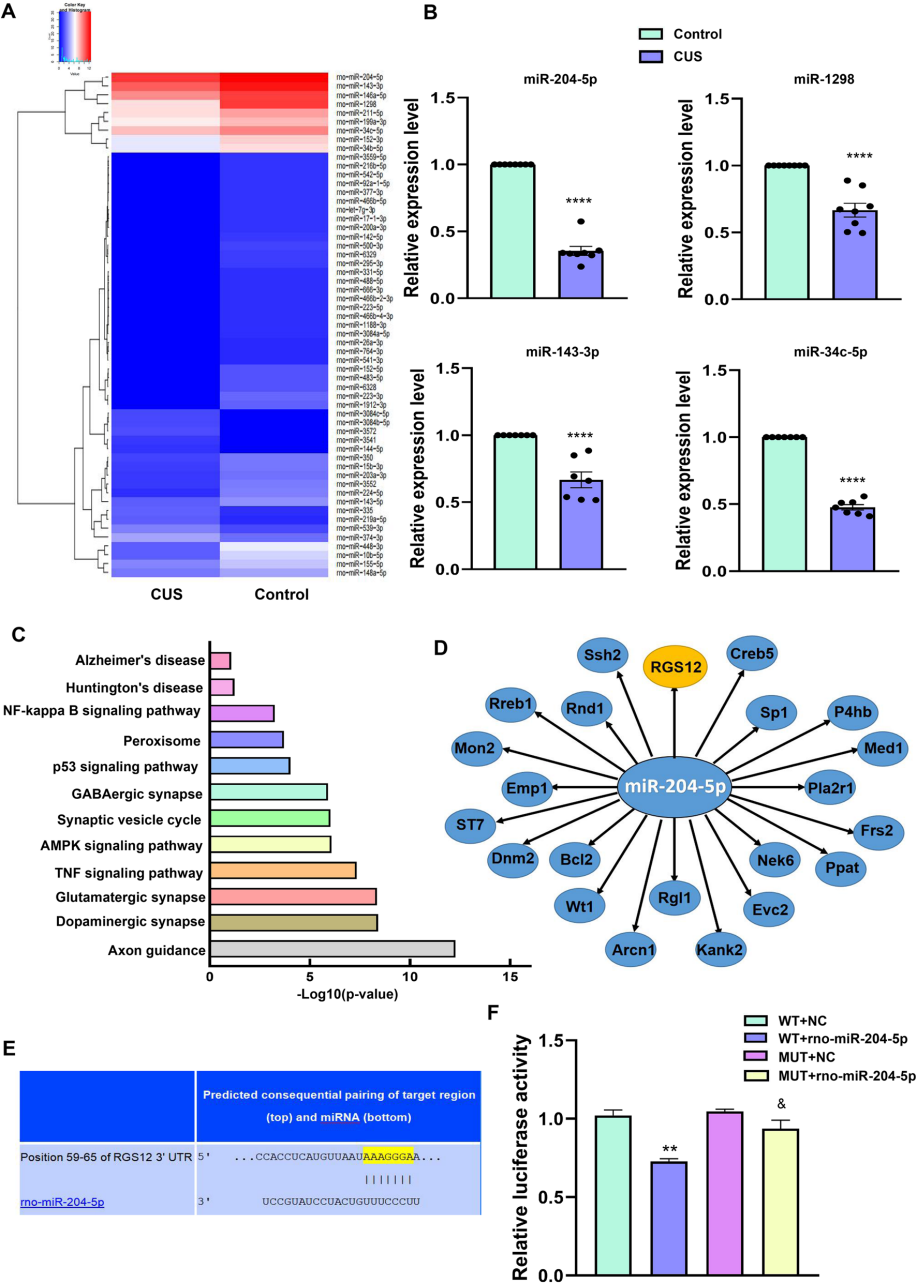
MiR-204-5p is reduced in the hippocampal DG region of CUS rats. **A** Heat map of differentially expressed miRNAs in hippocampal DG regions. **B** Relative expression levels of miR-204-5p, miR-1298, miR-143-3p and miR-34c-5p in the DG region (*N* = 7–8 per group). **P < 0.01, ****P < 0.0001, vs Control group. **C** Bar graphs represent magnitudes of significant correlations for miR-204-5p mediated signaling pathways as indicated by respective *P*-values (− log10 scaled). **D** Bioinformatical prediction of miR-204-5p target genes. **E** Predicted putative seed-matching sites between miR-204-5p and RGS12. **F** Luciferase reporter assay results as performed on 293Tcells to detect relative luciferase activities of WT and MUT RGS12 reporters (N = 3/group). **P < 0.01 vs WT + NC. ^&^*P* < 0.05 vs WT + rno-miR-204-5p. Data are presented as means ± SEMs. Student *t*-tests were employed for comparisons between the two groups. One-way ANOVA followed by Tukey’s post-hoc test was used for multiple comparisons involving > 2 groups

A total of 57 differentially expressed miRNAs with log2 (Fold Change) ≥ 1 and *P* values < 0.05 were identified within the hippocampal DG region of CUS versus control rats as determined using high throughput sequencing. As shown in Fig. 2A, the heat map generated showed results as obtained from a two-way hierarchical clustering of the samples and the differentially expressed miRNAs displaying relative expression levels as identified with high throughput sequencing. The differentially expressed miRNAs, consisting of miR-1298 (*P* < 0.0001), miR-204-5p (*P* < 0.0001), miR-143-3p (*P* < 0.0001) and miR-34c-5p (*P* < 0.0001), were then further validated with use of quantitative polymerase chain reaction (qPCR) analysis (Fig. 2B). The results, as presented in Fig. 2C, D, revealed that miR-204-5p was found to be associated with multiple signaling pathways (Fig. 2C) and involved with the regulation of multiple target genes (Fig. 2D). Notably, miR-204-5p was identified as being associated with oxidative phosphorylation signaling pathways. These findings directed us to concentrate our efforts on examining the role of miR-204-5p in depression. Among the multiple target genes regulated by miR-204-5p, we focused on RGS12. As this encoded protein regulates G protein-coupled receptor signaling cascades and is highly expressed in the central nervous system, it likely plays a key role in neuronal function and behavior. That miR-204-5p can directly regulate the expression of RGS12 proteins was revealed from luciferase reporter assay results demonstrating that miR-204-5p repressed reporter activity of the transcript containing the wild-type 3’-UTR of RGS12 (*P* < 0.05, Fig. 2E, F).

### MiR-204-5p expression within the DG region is associated with depression-like behaviors in CUS rats

**Fig. 3:**
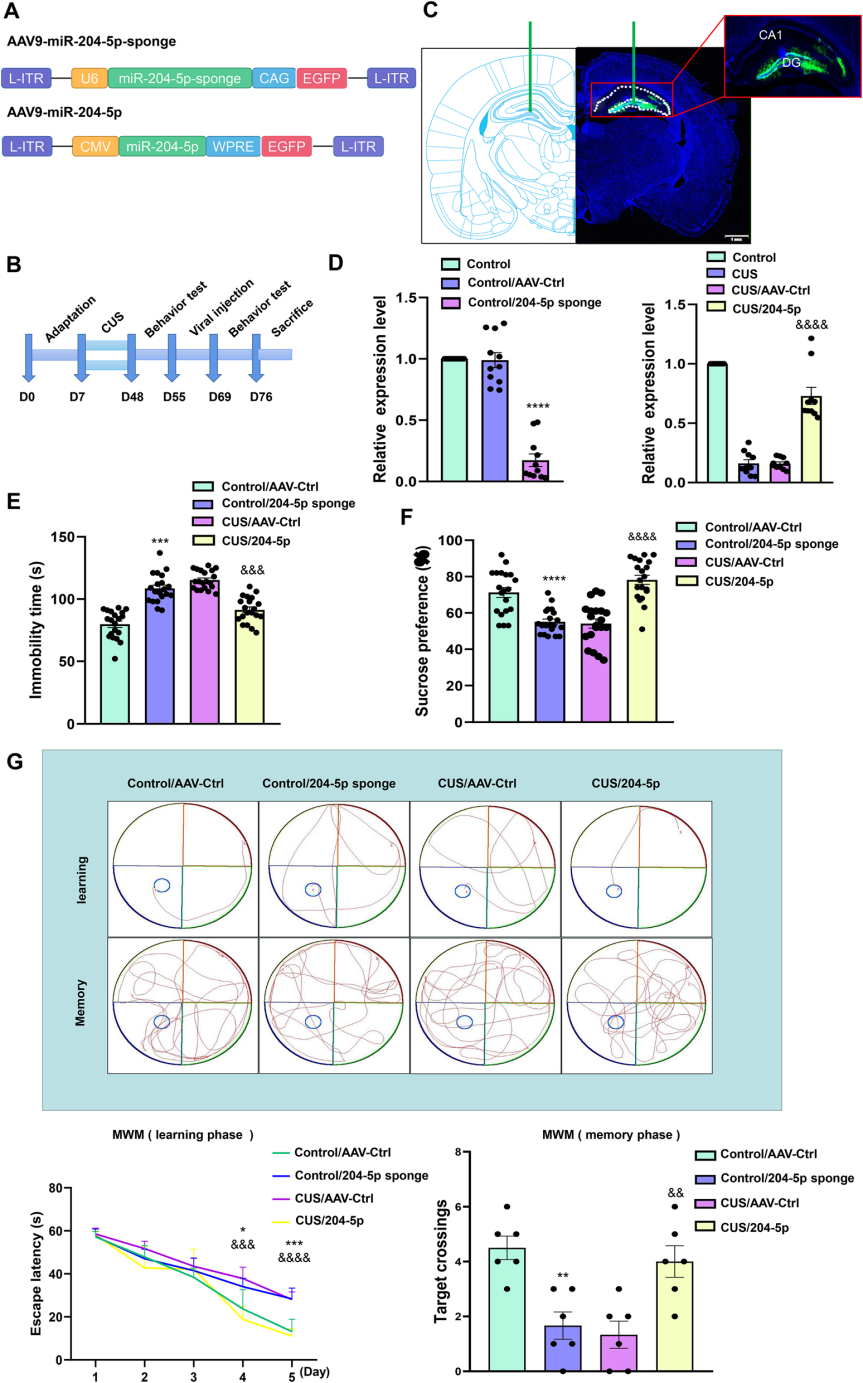
Effects of changes in miR-204-5p expression within the hippocampal DG region of normal and CUS rats. **A** Schematics of AAV vectors engineered to overexpress miR-204-5p, knock-down miR-204-5p and their corresponding controls. **B** Experimental paradigm for determining behavioral responses of rats infected with the virus. **C** Illustration of AAV viral infusion into the DG region. Scale bar is 1 mm. **D** The infection efficiency of AAV-miR-204-5p sponge and AAV-miR-204. Expression level of miR-204-5p in the DG region infected with AAV-204-5p or AAV-204-5p sponge (*N* = 10 per group). Behavioral responses in the (**E**) FST (*N* = 20 per group), (**F**) SPT (*N* = 20 per group) and (**G**) MWM of rats expressing various viral constructs within the DG region (N = 6 per group). *P < 0.05, **P < 0.01 ***P < 0.001, ****P < 0.0001, control + AAV-control vs control + AAV-miR-204-5p sponge; ^&^*P* < 0.05, ^&&^*P* < 0.01, ^&&&^*P* < 0.001, ^&&&&^*P* < 0.0001, CUS + AAV-control vs CUS + AAV-miR-204-5p. Data are presented as means ± SEMs. Student *t*-tests were employed for comparisons between the two groups. One-way ANOVA followed by Tukey’s post-hoc test was used for **D**. Two-way ANOVA was used for **E**–**F**

An AAV virus was constructed to overexpress miR-204-5p within the DG region of CUS rats or inhibit its expression in normal rats (Fig. 3A, B). Two weeks after virus injection, the observation of large amounts of green fluorescence within the DG region was considered indicative of a successful virus targeting injection (Fig. 3C), with a significant up-regulated expression of miR-204-5p being present in this region within CUS rats (*F*_(2, 30)_ = 107.3, *P* < 0.0001) and a significant down-regulated expression within normal rats (*F*_(3, 36)_ = 111.2, *P* < 0.0001) injected with the inhibition virus (Fig. 3D). CUS treatment increased their immobility times in the Forced Swimming Test (Fig. 3E), decreased the percentage of sucrose solution consumed (Fig. 3F) and decreased learning and memory abilities of these rats in the Morris Water Maze Test (Fig. 3G), effects which were essentially reversed after these CUS rats received an over-expression of miR-204-5p. In contrast, a down-regulation of miR-204-5p in normal rats led to behavioral responses indicative of a depression-like phenotype (FST: *F*_(3, 57)_ = 57.11, *P* < 0.0001. SPT: *F*_(3, 57)_ = 30.54, *P* < 0.0001. MWM: memory phase *F*_(3, 15)_ = 11.17, *P* < 0.001).

### MiR-204-5p knock-down in the DG region results in neuronal inflammation and oxidative stress in non-stressed normal rats

**Fig. 4:**
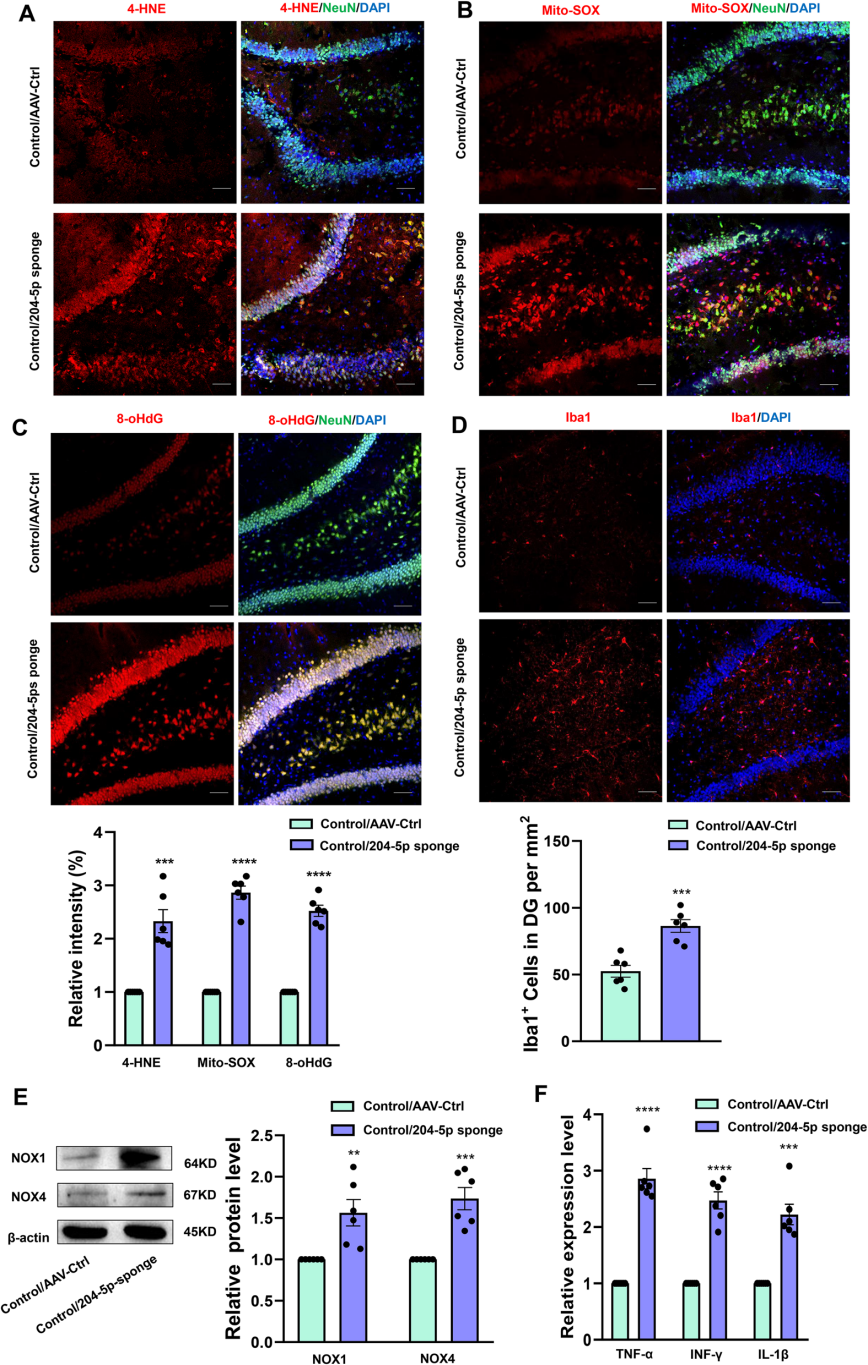
MiR-204-5p knock-down within the hippocampal DG region results in neuronal inflammation and oxidative stress in normal rats. **A** Representative confocal microscopic images showing expressions of 4-HNE in the DG region after AAV-miR-204-5p sponge infusion (*N* = 6 per group). Scale bar is 50 μm. **B** Representative images of Mito-Sox staining (red). Nuclei (blue) are stained with DAPI. Neurons (green) are stained with Anti-NeuN. Scale bar is 50 μm. (*N* = 6 per group). **C** Representative images of 8-oHdG staining (red). Scale bar is 50 μm. (*N* = 6 per group) **D** Immunofluorescent staining of Iba1 positive microglial cells within the DG region. Scale bar is 50 μm. (*N* = 6 per group) **E** Western bolt assays of protein expression levels of NOX1 and NOX4 within the DG region (*N* = 6 per group). **F** Q-PCR assays of mRNA expression levels of IL1β, TNF-α and IFN-γ within the DG region. Band intensities were normalized to GAPDH (*N* = 6 per group). **P < 0.01 ***P < 0.001, ****P < 0.0001, control + AAV-control vs control + AAV-miR-204-5p sponge. Data are presented as means ± SEMs. Student *t*-tests were employed for comparisons between the two groups

In order to examine the effects of reducing miR-204-5p expression within the hippocampal DG region of rats, oxidative stress levels were determined at this site. Knock-down of miR-204-5p significantly increased levels of 4-hydroxynonenal (4-HNE), a lipid peroxidation product and key mediator of oxidative stress-induced cell death (*P* < 0.001, Fig. 4A), and produced an increase in levels of superoxide within mitochondria, as measured by Mito-SOX Red fluorescent dye (*P* < 0.0001, Fig. 4B). In addition, as shown in Fig. 4C, the expression of 8-hydroxy deoxyguanosine (8-oHdG), a marker of oxidative DNA damage, within the DG region was significantly increased after a knock-down of miR-204-5p (*P* < 0.0001). Meanwhile, the protein expression of NOX1 (*P* < 0.01) and NOX4 (*P* < 0.001) were significantly increased after knockdown of miR-204-5p (Fig. 4E).

The excessive production of hippocampal DG ROS may be accompanied by inflammation and lead to cell damage. Results from our immunofluorescent assays showed that the number of Iba-1^+^ microglia within the DG region was significantly increased in rats with a knock-down of miR-204-5p (*P* < 0.001, Fig. 4D). As glial cell activation usually triggers secretions of cytokines, we next assessed expression levels of several critical pro-inflammatory cytokines. Following knock-down of miR-204-5p, mRNA expression levels of the pro-inflammatory cytokines TNF-α (*P* < 0.0001), IFN-γ (*P* < 0.0001) and IL-1β (*P* < 0.001), within the DG area were all significantly increased as compared to that in the non-stressed control group (Fig. 4F). There was no significant difference in neuroinflammation between the sham surgery group, the AAV virus injection group and control group (Additional file 3: Fig. S1).

### MiR-204-5p overexpression in the DG region alleviates neuronal inflammation and oxidative stress in CUS rats

**Fig. 5:**
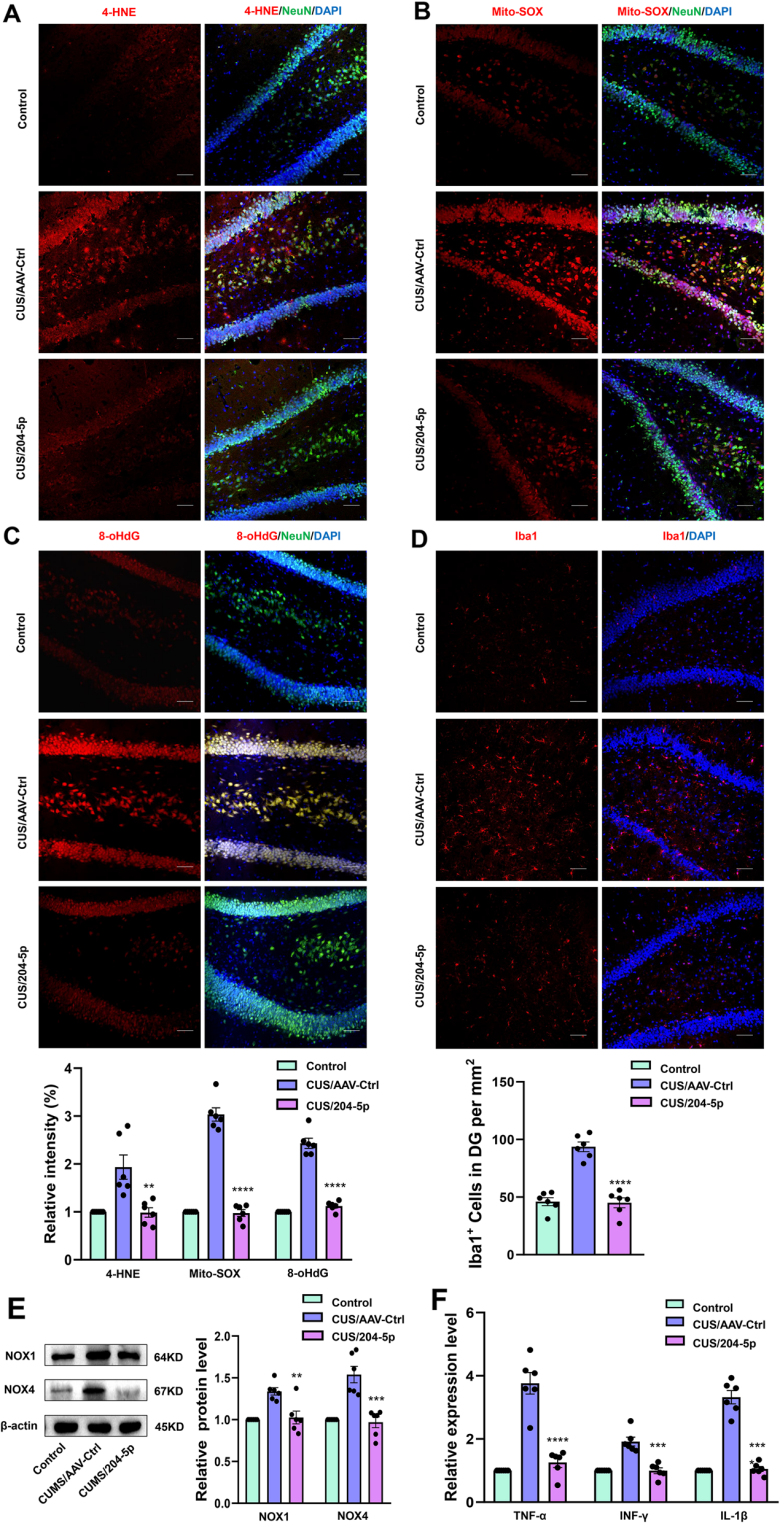
MiR-204-5p overexpression within the hippocampal DG region alleviates neuronal inflammation and oxidative stress in CUS rats. **A** Representative confocal microscopic images showing expressions of 4-HNE in the DG region after AAV-miR-204-5p infusion (*N* = 6 per group). Scale bar is 50 μm. **B** Representative images of Mito-Sox staining (red). Nuclei (blue) are stained with DAPI. Neurons (green) are stained with Anti-NeuN. Scale bar is 50 μm. (*N* = 6 per group) **C** Representative confocal microscopic images of immunostainings for 8-oHdG in the DG region. Scale bar = 50 μm. (*N* = 6 per group) **D** Immunofluorescent staining of Iba1 positive microglial cells within the DG region. Scale bar is 50 μm. (*N* = 6 per group) **E** Western bolt assays of protein expression levels of NOX1 and NOX4 within the DG region *(N* = 6 per group). **F** Q-PCR showing mRNA levels of pro-inflammatory cytokines, Band intensities were normalized to GAPDH. *N* = 6 per group, ^****^P < 0.0001, CUS + AAV-control vs CUS + AAV-miR-204-5p. Data are presented as means ± SEMs. Student *t*-tests were employed for comparisons between the two groups. One-way ANOVA followed by Tukey’s post-hoc test was used for multiple comparisons involving > 2 groups

In this experiment, AAV-miR-204-5p was infused bilaterally within the DG region of CUS rats to significantly increase miR-204-5p expression. In response to this treatment, expression levels of oxidation-related factors (*F*_(2, 15)_ = 12.16, *P* < 0.01, Fig. 5A), DNA oxidative damage markers (*F*_(2, 15)_ = 17.9, *P* < 0.0001, Fig. 5B), mitochondrial ROS levels (*F*_(2, 15)_ = 15.7, *P* < 0.0001, Fig. 5C) and the protein expression of NOX1 (*F*_(2, 15)_ = 13.45, *P* < 0.01) and NOX4 (*F*_(2, 15)_ = 22.31, *P* < 0.001) (Fig. 5E) all showed reductions in oxidative stress within the DG region of these CUS rats. Meanwhile, after miR-204-5p was overexpressed, the activities of a number of antioxidant enzymes in the hippocampal DG region of CUS rats was enhanced (SOD: *F*_(2, 15)_ = 19.84, *P* < 0.0001, T-AOC: *F*_(2, 15)_ = 43.57, *P* < 0.0001), the levels of the oxidative stress products were decreased (MDA: *F*_(2, 15)_ = 31.22, *P* < 0.0001, LDH: *F*_(2, 15)_ = 21.10, *P* < 0.0001) and the mRNA expression of NOX1 (*F*_(2, 15)_ = 49.97, *P* < 0.0001) and NOX4 (*F*_(2, 15)_ = 176.8, *P* < 0.0001) were decreased (Additional file 4: Fig. S2, Additional file 5). The findings of significant reductions in levels of pro-inflammatory cytokines (TNF-α: *F*_(2, 15)_ = 49.96, *P* < 0.0001; IFN-γ: *F*_(2, 15)_ = 29.69, *P* < 0.001; IL-1β: *F*_(2, 15)_ = 52.95, *P* < 0.0001, Fig. 5F) and reductions in the number of activated microglia (*F*_(2, 18)_ = 24.2, *P* < 0.0001, Fig. 5D) provide further evidence that miR-204-5p overexpression was effective in reducing the degree of neuroinflammation in these CUS rats. These results indicate that an overexpression of miR-204-5P can diminish the oxidative stress and inflammation present within the DG region of CUS rats. Therefore, the loss of miR-204-5p within a discrete region of the brain, namely the hippocampal DG area, may result in neuronal dysfunctions involved with the induction of depression-like behaviors in CUS rats.

### MIR-204-5p inhibits oxidative stress within the DG region possibly via the RGS12/Nrf2/NF-κB pathway

**Fig. 6:**
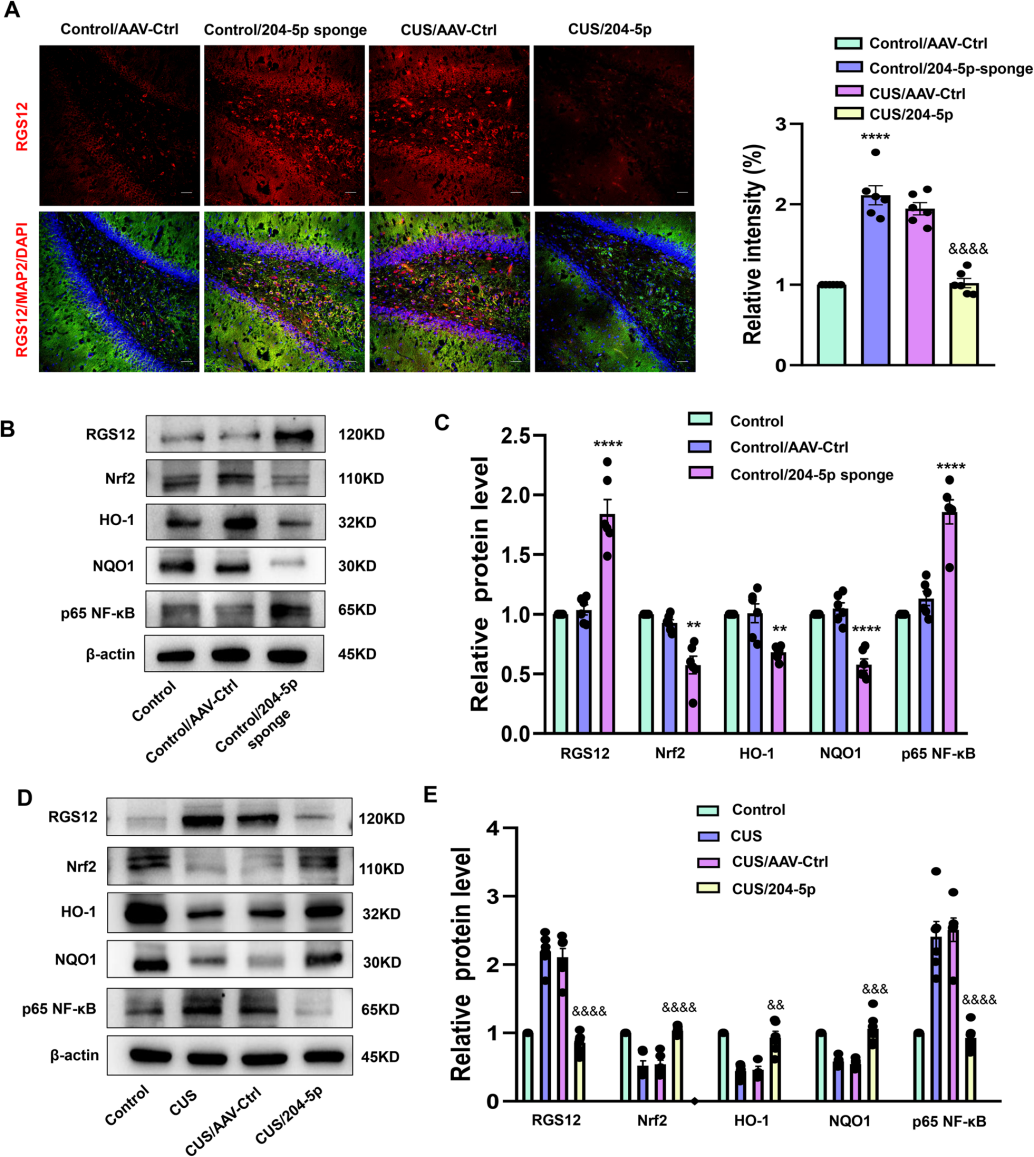
MiR-204-5p inhibits the occurrence of oxidative stress within the hippocampal DG region via the RGS12/Nrf2-NF-κB pathway. **A** Representative images of RGS12 (red) and MAP2 (green) staining. Nuclei (blue) are stained with DAPI. Scale bar is 50 μm. (*N* = 6 per group) **B**, **C** Representative western blot images showing relative protein levels of RGS12, Nrf2, NQO1, HO-1 and NF-κB p65 in AAV-miR-204-5p sponge virus infected DGs (N = 6 per group). (D-E) Representative western blot images showing relative protein levels in AAV-miR-204-5p virus infected DGs (*N* = 6 per group). **P < 0.01 ***P < 0.001, ****P < 0.0001, control + AAV-control vs control + AAV-miR-204-5p sponge; ^&&^*P* < 0.01, ^&&&^*P* < 0.001, ^&&&&^*P* < 0.0001, CUS + AAV-control vs CUS + AAV-miR-204-5p. Data are presented as means ± SEMs. Student *t*-tests were employed for comparisons between the two groups. Two-way ANOVA was used for Fig. 6A. One-way ANOVA followed by Tukey’s post-hoc test was used for **C** and **E**

We next investigated some of the downstream molecular mechanisms of miR-204-5p as involved with regulating oxidative stress in the DG area. Given that miR-204-5p directly regulates RGS12 expression, we determined the protein levels of RGS12 in the DG region of these AAV-miR-204-5p virus treated rats. Results from our immunofluorescent assay showed that RGS12 expression was significantly decreased in AAV-miR-204-5p virus treated CUS rats, while the protein expression of RGS12 was increased following knock-down of miR-204-5p in normal rats (*F*_(3, 15)_ = 79.58, *P* < 0.0001, Fig. 6A). Western blot assay results revealed that protein expressions of RGS12 (*F*_(2, 15)_ = 40.86, *P* < 0.0001) and NF-κB p65 (*F*_(2, 15)_ = 47.09, *P* < 0.0001) were significantly increased after miR-204-5p expression was reduced in normal rats, while levels of Nrf2 (*F*_(2, 15)_ = 25.10, *P* < 0.01), HO-1 (*F*_(2, 15)_ = 15.41, *P* < 0.01) and NQO1 (*F*_(2, 15)_ = 43.65, *P* < 0.0001) were all notably decreased (Fig. 6B, C). Within CUS rats, expressions of Nrf2 (*F*_(3, 20)_ = 34.60, *P* < 0.0001) and HO-1 (*F*_(3, 20)_ = 29.50, *P* < 0.01), as well as levels of NQO1 (*F*_(3, 20)_ = 29.90, *P* < 0.001) were all significantly increased within DG areas following AAV-miR-204-5p injections (Fig. 6D, E). These results provide the evidence indicating that miR-204-5p affected oxidative stress in DG neurons possibly via the RGS12/Nrf2 /NF-κB pathway.

### MiR-204-5p ameliorated dysregulation of synaptic plasticity in CUS rats

**Fig. 7:**
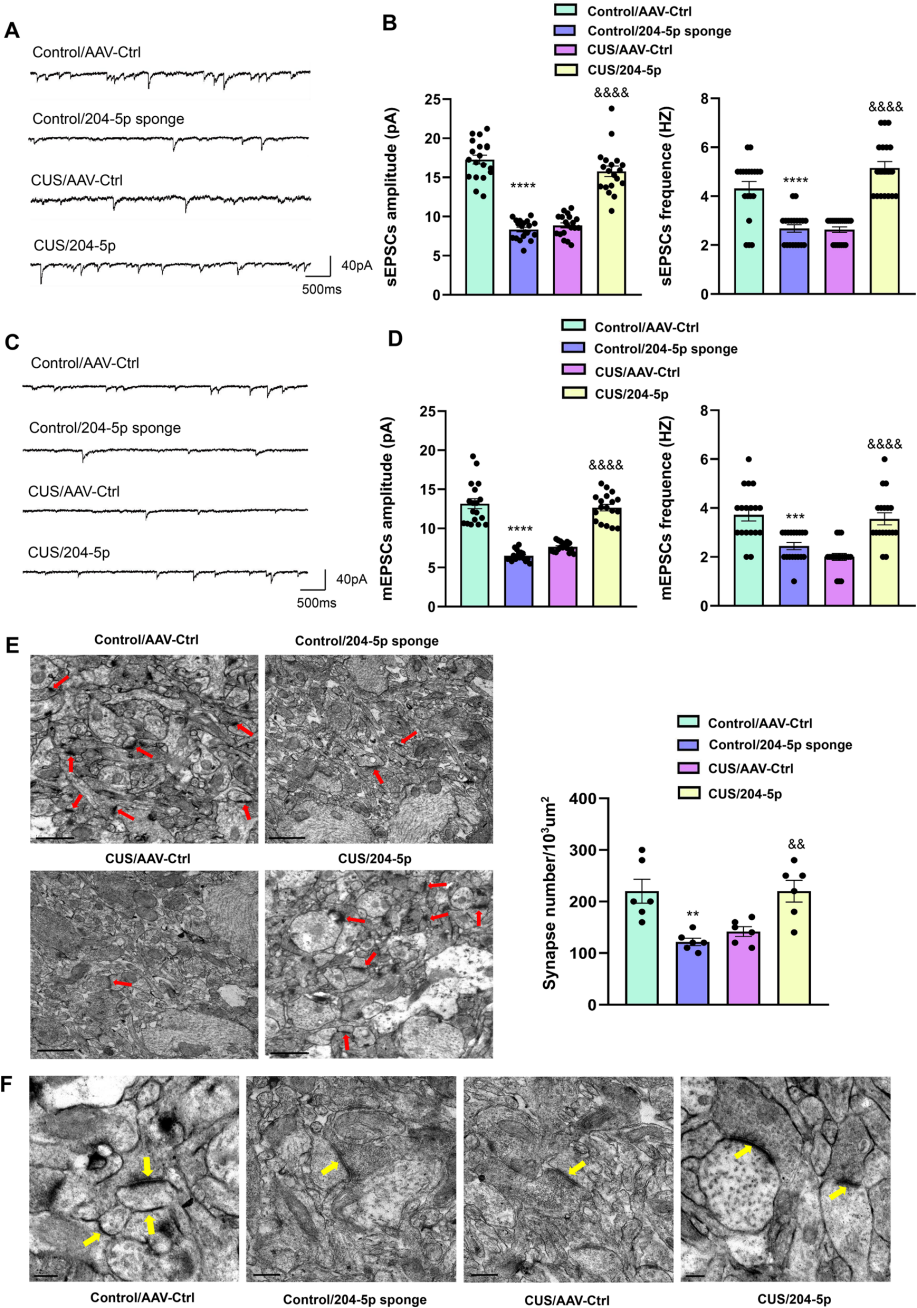
MiR-204-5p regulates synaptic plasticity of DG neurons in rats. **A** Representative traces of sEPSCs in DG neurons of rats infected with the virus. **B** Cumulative fraction plots of sEPSCs amplitudes and frequencies. **C** Cumulative fraction plots of mEPSCs amplitudes and frequencies. **D** Representative traces of mEPSCs in DG neurons of rats infected with the virus. *N* = 18 neurons from six rats in each group. **E** Representative electron micrographs and summary of data showing the number of synapsis. Arrows indicate neuronal synapses. Scale bar is 1 μm. (*N* = 6 per group). **F** Representative electron micrograph of DG neurons in rats from each group (*N* = 6 per group). Arrows indicate neuronal synapses. Scale bar is 0.2 μm. **P < 0.01 ***P < 0.001, ****P < 0.0001, control + AAV-control vs control + AAV-miR-204-5p sponge; ^&&^*P* < 0.01, ^&&&&^*P* < 0.0001, CUS + AAV-control vs CUS + AAV-miR-204-5p. Data are presented as means ± SEMs. Student *t*-tests were employed for comparisons between the two groups. Two-way ANOVA was used for multiple comparisons involving > 2 groups

As oxidative stress can promote additional pathological processes during the onset of depression, we examined the effects of miR-204-5p on synaptic morphology and function in rats. With use of whole-cell patch-clamp recordings of brain slices, we observed that the frequencies and peaks of miniature excitatory postsynaptic currents and spontaneous excitatory postsynaptic currents were increased within the hippocampal DG region of CUS rats after up-regulation of miR-204-5p. Conversely, with a decrease in the expression of DG miR-204-5p in normal rats there was a reduction in the frequencies and peak values of these miniature excitatory postsynaptic currents and spontaneous excitatory postsynaptic currents, indicating impairments in synaptic transmission function (sEPSC: amplitude *F*_(3, 54)_ = 95.36, *P* < 0.0001; frequency *F*_(3, 54)_ = 31.14, *P* < 0.0001. mEPSC: amplitude *F*_(3, 51)_ = 85.13, *P* < 0.0001; frequency *F*_(3, 51)_ = 16.38, *P* < 0.0001, Fig. 7A–D). Observations as obtained with use of transmission electron microscopy also showed that the number of hippocampal DG synapses in rats after knocking-down of miR-204-5p was significantly reduced, the bouton and mushroom spine volume was decreased, as well as the decreased post-synaptic density (PSD) in the synapses. In contrast, overexpression of miR-204-5p within the DG region of CUS rats was associated with increased numbers of synapses *F*_(3, 15)_ = 12.04, *P* < 0.001, Fig. 7E, F). These results indicate that miR-204-5p is involved in regulating the morphological and functional condition of neuronal synapses that occur with depression.

## Discussion

Of late, the relationship between oxidative stress damage and neurodegenerative/neuropsychiatric disorders as well as the potential molecular mechanisms involved have aroused great interest among researchers [21]. Findings from our laboratory have provided the first evidence that miR-204-5p is involved in regulating oxidative stress as associated with the pathological processes of depression. MiR-204-5p may activates the Nrf2/NF-kB pathway by targeting the downstream gene, RGS12, to regulate oxidative stress and neuroinflammation in the hippocampal DG region of rats. An overexpression of miR-204-5p attenuates depression-like behaviors observed in CUS rats, as well as the neuronal and synaptic plasticity damage resulting from oxidative stress in this rat model of depression. In summary, our results indicate that miR-204-5p can function as a critical target possessing a potential for regulating oxidative stress during the pathogenesis of depression.

Oxidative stress is considered as one of the important regulators of aging and various neurological diseases [22].Results from clinical studies have also indicated that a variety of highly expressed oxidative stress markers are present in the brains of patients with depression [23]. We have established that CUS can effectively induce depression-like behaviors in rats and found that substantial amounts of ROS are present in the vmPFC, hippocampal CA1 and DG regions in this rat model of depression. Moreover, NADPH oxidase is increased, while antioxidant enzyme activity decreased in these CUS rats, which indicates that the occurrence of depression is accompanied by oxidative stress damage. While the hippocampus, in particular the hippocampal DG region, which is thought to be involved in mood and cognitive regulation during the onset of depression, has undergone severe disruption. Moreover, in clinical trials, small volume DG regions in depressed patients have been demonstrated to resemble depressed animals [24, 25]. Therefore, in the present study, we mainly focused on exploring the causes of oxidative damage in hippocampal DG region of CUS model rats. Increasing evidence from recent studies has revealed the importance of miRNAs as biomarkers for specific central nervous system diseases. Some of these miRNAs are involved with regulating oxidative stress-related pathways and participate in the development of various neurological diseases. As an approach to identify potential molecular targets for regulating oxidative stress and related mechanisms in depression, we performed a miRNA tissue transcriptome sequencing analysis in the hippocampal DG region of CUS and normal rats. Differentially expressed miRNAs were then screened from the RNA-sequencing results and a KEEG database analysis was performed. Based on KEEG analysis of miR-204-5p and its downstream predictive target genes and previous literature reports, it is concluded that miR-204-5p is associated with a variety of diseases related to the central nervous system [26]. Findings from previous reports revealed that miR-204-5p is involved in multiple signaling pathways and results from related studies have shown that miR-204-5p is involved with oxidative stress-related pathways, in specific to the oxidative capacity of mitochondria as demonstrated in vivo [27–29]. Accordingly, the potential role of miR-204-5p in depression became the focus of our research in this report.

Our current results reveal that miR-204-5p expression was significantly down-regulated in CUS rats. Such findings then persuaded us to examine the effects of altering miR-204-5p expression levels within the hippocampal DG region of CUS and normal rats. When the expression of miR-204-5p in the DG region was reduced in normal rats, depression-like behaviors and decreases in cognition and learning ability were observed. In addition, the DG region of these rats contained considerable amounts of ROS in the mitochondria of neurons and the oxidative stress marker, 4-HNE, was also significantly increased. Conversely, an overexpression of miR-204-5p in the DG region of CUS rats reduced the display of depression-like behaviors and alleviated the oxidative stress injury in their brain. As the complementary relationship between oxidative stress and inflammation in the pathogenesis of depression is well established, the excessive amounts of ROS as produced during the oxidation process in these CUS rats can result in neuronal damage and aggravate neuroinflammation [30]. Given that miR-204-5p regulates oxidative stress, is accompanied by increases in pro-inflammatory cytokines, and activates a large number of microglia in the DG region, the potential significance of its role in depression should be readily appreciated.

To further explore some of the possible molecular mechanisms of miR-204-5p as involved in regulating oxidative stress processes in depression we utilized database screening and dual luciferase reporter gene analysis. From this analysis, a member of the regulator of G protein signaling (RGS) gene family, RGS12, was identified as a candidate for further investigation. There exists a sizeable amount of evidence for focusing on RGS proteins as related to neurological disorders. Notably, RGS proteins are differentially expressed in most brain regions, are significantly up- or down-regulated during pathophysiology [31, 32]. In addition, RGS proteins can regulate the physiological effects of many neurotransmitters, hormones and other signaling molecules, making them attractive targets for drug therapies in central nervous system diseases [33, 34]. As related to depression, the RGS family members, RGS2, RGS4, RGS6 and RGS8 have all surfaced as being involved with regulating the manifestation of depression or play role as antidepressants through their capacity to activate different downstream molecular mechanisms [35]. As the largest protein in the RGS family, RGS12, has a special multi-domain structure. Such a structure may serve as a basis for RGS12’s involvement in the occurrence of a number of diseases through its ability to trigger a variety of signaling pathways [36]. It has been reported that RGS12 promotes the production of reactive oxygen species and osteoclasts by inhibiting Nrf2 [37]. In our current study, we found that the decreased expression of miR-204-5p in the hippocampal DG region of CUS rats was accompanied with increases in the expression of the targeting molecule RGS12, while the expression of Nrf2 and its downstream targets, NQO1 and HO-1, were significantly decreased. Contrarily, when miR-204-5p is overexpressed, RGS12 expression increases. Oxidative stress can trigger neuronal inflammation by altering protein conformations and destroying nucleic acids. In related studies, it has been reported that RGS12 regulates the inflammation of arthritis by activating NF-κB to generate a feedback loop [38]. Therefore, we propose that as a result of miR-204-5p targeting RGS12 to regulate the occurrence of depression, the increase in NF-κB p65 is not only related to neuroinflammation resulting from oxidative stress injury, but is also related to the activation of NF-κB in response to the increase of RGS12.

The dysfunction and loss of synaptic connections are closely related to cognitive impairments in depression [39]. In the Morris water maze test, we found that CUS rats showed improved learning and cognitive ability when miR-204-5p was overexpressed. Related to these behavioral results, were increases in frequencies and peak values of miniature excitatory postsynaptic currents in the hippocampal DG region of CUS rats after miR-204-5p was upregulated, as determined from results of whole-cell patch-clamp recordings. Moreover, with use of transmission electron microscopy, we observed that the number of synapses in the hippocampal DG region of normal rats with a down-regulation of miR-204-5p was significantly reduced and the morphological structure of remaining synapses showed clear evidence of damage. Large amounts of ROS as produced by oxidative stress can result in a certain degree of damage to neuronal cell membranes and mitochondria. As mitochondria regulate Ca^2+^ and mediate the conduction of redox signals and energy metabolism, their function and motility are closely related to the growth of synapses and synaptic connections [40]. Therefore, the cell damage caused by oxidative stress may further affect synaptic plasticity [8]. Based on the results of this study, we believe that a potential mechanism through which miR-204-5p affects synaptic plasticity is related to its regulation of oxidative stress via the targeting of RGS12. The miR-204-5p target gene, RGS12, is expressed in dendritic and postsynaptic membranes at the subcellular level [41], and there is a PDZ domain in its special multi-domain structure. This PDZ domain could control the composition and structure of synaptic proteins and play an important role in the dynamic transport of synaptic proteins [36]. RGS14, which has a very similar structure to that of RGS12, has also been reported to inhibit hippocampal CA2 neurons and synaptic plasticity at this site [42]. Therefore, we hypothesized that the potential mechanism of miR-204-5p involved in regulating synaptic plasticity might be mediated by targeting the expression level of RGS12.

It should be pointed out that this study focused on exploring the regulatory effects of miR-204-5p on the behavioral and neurobiological changes in the hippocampal DG region of CUS model rats. Results of this study showed that miR-204-5p significantly regulates oxidative stress in hippocampus DG region via targeting RGS12 signaling pathway. However, miR-204-5p has several downstream target genes, therefore, whether other downstream target genes are involved in the regulation of oxidative stress is also worthy of further investigation. Second, the rats used in the present study was in the adolescent stage, so the high sensitivity of the adolescent brain to stress may also be a contributing factor to the apparent pathological phenomena observed. In addition, rats sustained iced-swimming stimulation in CUS procedure which may make them adapt to swim, so the forced swimming test used to evaluate depression-like behavior may not sufficient. Finally, the previous literature indicated that miR-204-5p expression was elevated in the anterior cingulate cortex and habenula in MDD patients [43]. Depression is a complex disease which involves potential interactions between gene–environment and gene–sex factors, so its related molecules may have different changes in different brain regions. In the animal model of depression used in this study, we observed decreased expression of miR-204-5p in the DG and CA1 region of the hippocampus which caused enhanced oxidative stress damage in depression. However, the detailed mechanisms of miR-204-5p pathway in the development of depression remain to be further explored.

## Conclusion

In conclusion, the findings presented in this report demonstrate that miR-204-5p can be used as a potential therapeutic target for the treatment of depression. MiR-204-5p’s downstream target, RGS12, possibly regulates Nrf2/NF-κB to promote oxidative stress and neuroinflammation. Moreover, miR-204-5p also appears to be involved in the regulation of synaptic plasticity within the hippocampal DG region. While our data provide relatively strong support for these hypothesized mechanisms, further research will be required to corroborate these findings. In summary, the results of this study, reveal a new targeted molecule and some of the potential mechanism of oxidative stress that may be involved in the pathogenesis of depression. Such findings provide a bases for a new strategy in the treatment of depression.

## Supplementary Information

**Additional file 1: Table S1.** Primer sequences of target genes used for Reverse transcription PCR in this study.

**Additional file 2: Table S2.** The up-regulated and down-regulated MicroRNAs with significant differences in expression ranked by fold changes in microarray.

**Additional file 3: Fig. S1.** Effects of sham operation and AAV injection on rats. (A) Immunofluorescent staining of Iba1 positive microglial cells within the DG region. Scale bar is 50 μm. (N = 6 per group). (B) Q-PCR analysis of IL-1βand IL-6 mRNA levels of each group. Band intensities were normalized to GAPDH (N = 6 per group). NS > 0.05. Data are presented as means ± SEMs. Student *t*-tests were employed for comparisons between the two groups.

**Additional file 4: Fig. S2.** MiR-204-5p overexpression within the hippocampal DG region alleviates oxidative stress in CUS rats. (A-B) Activity of antioxidant enzymes SOD and T-AOC. Contents of MDA and LDH were analyzed and levels were normalized to total protein content (N = 6 per group). (C) Q-PCR analysis of NOX1 and NOX4 mRNA levels of each group. Band intensities were normalized to GAPDH (N = 6 per group). **P < 0.01, ***P < 0.001, ****P < 0.0001, CUS + AAV-control vs CUS + AAV-miR-204-5p. Data are presented as means ± SEMs. Student *t*-tests were employed for comparisons between the two groups.

**Additional file 5.** Figures S1, S2 legends.

## Data Availability

The data that support the findings of this study are available from the corresponding author upon reasonable request.

## Abbreviations

MDD: Major depressive disorder
CUS: Chronic unpredictable stress
CNS: Central nervous system
DG: Dentate gyrus
vmPFC: Ventral medial prefrontal cortex
RGSG: Regulator of G protein signaling
miRNAs: MicroRNAs
KEEG: Kyoto Encyclopedia of Genes and Genomes
FST: Forced swimming test
SPT: Sucrose preference test
MWM: Morris water maze
Q-PCR: Real-time quantitative PCR
WT: Wild type
MUT: Mutant type
PFA: Paraformaldehyde
TEM: Transmission electron microscopy
ACSF: Artificial cerebral spinal fluid
DHE: Dihydroethidium
ANOVA: Analysis of variance
SOD: Superoxide dismutase
T-AOC: Total antioxidant capacity
MDA: Malondialdehyde
LDH: Lactate dehydrogenase
ROS: Reactive oxygen species
NOX: NADPH oxidases
PSD: Post-synaptic density
