# Supplementary material for: MicroRNA-204-5p reduction in rat hippocampus contributes to stress-induced pathology via targeting RGS12 signaling pathway
Source: J Neuroinflammation. 2021 Oct 21;18:243. doi: 10.1186/s12974-021-02299-5 (PMC8532383; doi:10.1186/s12974-021-02299-5)
Supplement: Supplementary file 1 — Additional file 1: Table S1. Primer sequences of target genes used for Reverse transcription PCR in this study. [file 12974_2021_2299_MOESM1_ESM.docx]

**Table S1. Primer sequences of target genes used for Reverse transcription PCR in this study**

| Gene | Forword (5’→3’) | Reverse (5’→3’) |
| --- | --- | --- |
| IL-1β | AAG ATG AAG GGC TGC TTC CAA ACC | ATA CTG CCT GCC TGA AGC TCT TGT |
| IFN-γ | ATT CAT GAG CAT CGC CAA GTT C | TGA CAG CTG GTG AAT CAC TCT GAT |
| TNF-α | TGA TCG GTC CCA ACA AGG A | TGC TTG GTG GTT TGC TAC GA |
| NOX1 | CCT GAA GGA TCC CAT CAG AGA | TGG AGG TCT GGA GCC TCT TA |
| NOX4 | CCG GAC AGT CCT GGC TTA TC | TTG AGG GCA TTC ACC AAG TG |
| GAPDH | AGT GCC AGC CTC GTC TCA TA | GGT AAC CAG GCG TCC GAT AC |


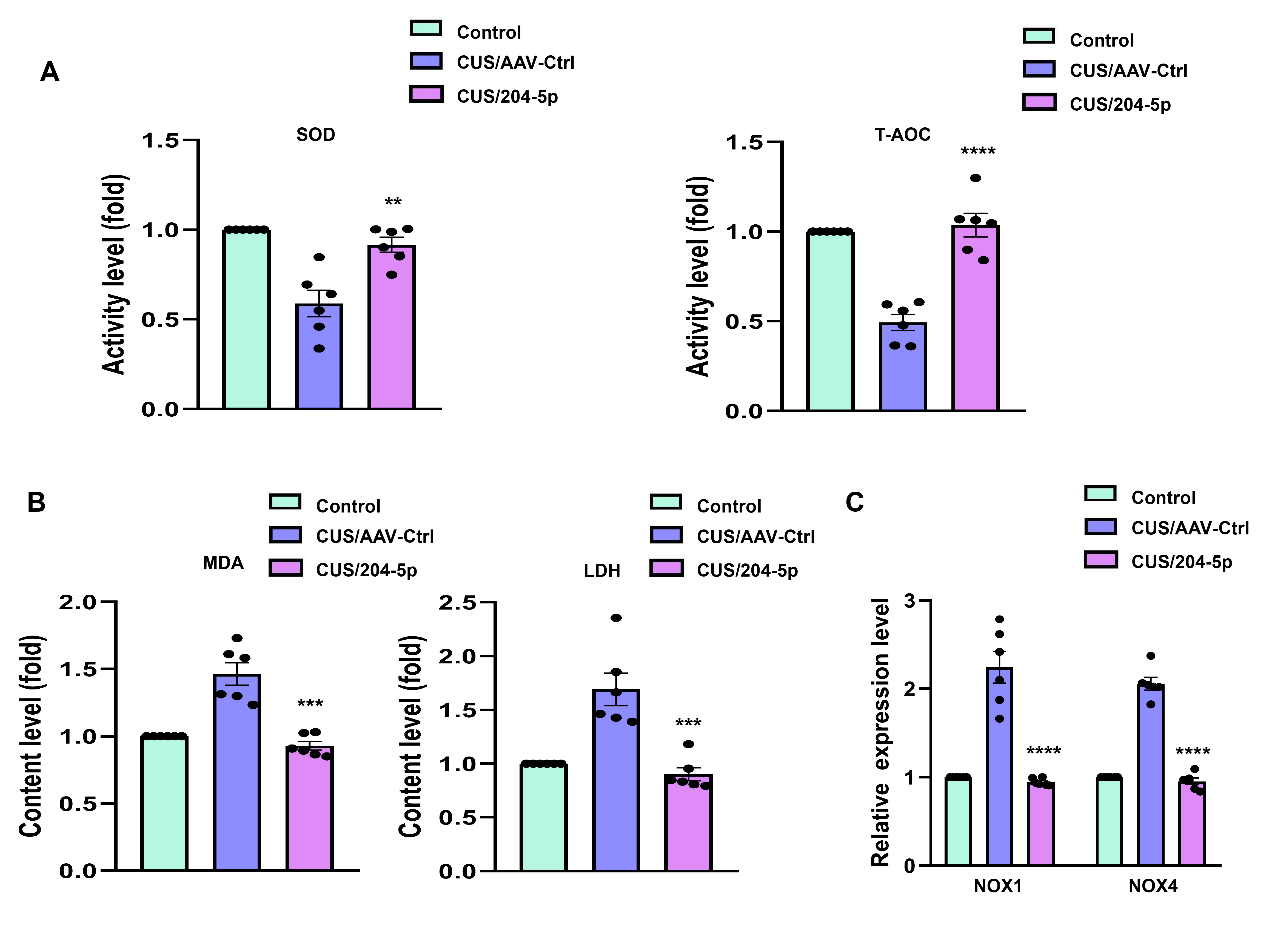


**Supplementary Figure S1. Overexpression of miR-204-5p in the hippocampal DG region can alleviates oxidative stress in CUS rats. (A)** Activity of antioxidant enzymes SOD and T-AOC (N=6 per group). **(B)** Contents of MDA and LDH were analyzed and levels were normalized to total protein content (N=6 per group). **(C)**  Q-PCR analysis of NOX1 and NOX4 mRNA levels of each group. Band intensities were normalized to GAPDH (N=6 per group). **P <0.01, ***P <0.001, ****P <0.0001, CUS+ AAV-control vs CUS+ AAV-miR-204-5p. Data are presented as means ± SEMs. Student t-tests were employed for comparisons between the two groups.
