# Supplementary material for: MicroRNA-204-5p reduction in rat hippocampus contributes to stress-induced pathology via targeting RGS12 signaling pathway
Source: J Neuroinflammation. 2021 Oct 21;18:243. doi: 10.1186/s12974-021-02299-5 (PMC8532383; doi:10.1186/s12974-021-02299-5)
Supplement: Supplementary file 2 — Additional file 2: Table S2. The up-regulated and down-regulated MicroRNAs with significant differences in expression ranked by fold changes in microarray. [file 12974_2021_2299_MOESM2_ESM.docx]

**Table S2. The up-regulated and down-regulated MicroRNAs with significant differences in expression ranked by fold changes in microarray.**

Note: P-value: P-value calculated from paired t-test. FDR: FDR is calculated from Benjamini Hochberg FDR. log2(fold change): Logarithmic expression differential multiples based on 2. Significance-Lab: If | log2 (a Fold Change) | ≥ 1 and P - value< 0.01, the significance-label is **, If | log2 (a Fold Change) | ≥ 1 and P - value< 0.05, the significance-label is *.

| **miRNA_ID** | **log2(fold change)** | **P-value** | **FDR** | **Significance** |
| --- | --- | --- | --- | --- |
| **Down-regulated MicroRNAs** | | | | |
| rno-miR-223-3p | 8.983 | 1.45E-06 | 0.000368147 | ** |
| rno-miR-1912-3p | 8.8918 | 2.45E-06 | 0.000468053 | ** |
| rno-miR-483-5p | 8.4136 | 2.82E-05 | 0.003082965 | ** |
| rno-miR-152-5p | 8.4136 | 2.82E-05 | 0.003082965 | ** |
| rno-miR-6328 | 8.3219 | 3.57E-05 | 0.003407488 | ** |
| rno-miR-500-3p | 7.7616 | 0.000390825 | 0.024882502 | ** |
| rno-miR-6329 | 7.6935 | 0.000530405 | 0.031171486 | ** |
| rno-miR-295-3p | 7.6147 | 0.000726851 | 0.039665304 | ** |
| rno-miR-142-5p | 7.4594 | 0.001408973 | 0.067278458 | ** |
| rno-miR-466b-5p | 7.2854 | 0.002863891 | 0.084154325 | ** |
| rno-miR-216b-5p | 7.2761 | 0.002863891 | 0.084154325 | ** |
| rno-miR-3559-5p | 7.2761 | 0.002863891 | 0.084154325 | ** |
| rno-miR-542-5p | 7.2761 | 0.002863891 | 0.084154325 | ** |
| rno-miR-92a-1-5p | 7.2761 | 0.002863891 | 0.084154325 | ** |
| rno-miR-377-3p | 7.2761 | 0.002863891 | 0.084154325 | ** |
| rno-miR-17-1-3p | 7.1799 | 0.002863891 | 0.084154325 | ** |
| rno-let-7g-3p | 7.1799 | 0.002863891 | 0.084154325 | ** |
| rno-miR-200a-3p | 7.1799 | 0.002863891 | 0.084154325 | ** |
| rno-miR-3084a-5p | 7.0661 | 0.004165659 | 0.113662984 | ** |
| rno-miR-488-5p | 6.9542 | 0.006149306 | 0.123633421 | ** |
| rno-miR-331-5p | 6.9542 | 0.006149306 | 0.123633421 | ** |
| rno-miR-666-3p | 6.9542 | 0.006149306 | 0.123633421 | ** |
| rno-miR-466b-2-3p | 6.9542 | 0.006149306 | 0.123633421 | ** |
| rno-miR-223-5p | 6.9542 | 0.006149306 | 0.123633421 | ** |
| rno-miR-466b-4-3p | 6.9542 | 0.006149306 | 0.123633421 | ** |
| rno-miR-1188-3p | 6.9542 | 0.006149306 | 0.123633421 | ** |
| rno-miR-764-3p | 6.8329 | 0.009223959 | 0.17188061 | ** |
| rno-miR-26a-3p | 6.8329 | 0.009223959 | 0.17188061 | ** |
| rno-miR-541-3p | 6.6865 | 0.014078675 | 0.256097801 | * |
| rno-miR-1298 | 4.0736 | 3.22E-08 | 2.46E-05 | ** |
| rno-miR-448-3p | 3.8298 | 5.33E-07 | 0.000203694 | ** |
| rno-miR-10b-5p | 3.2338 | 1.42E-05 | 0.002172949 | ** |
| rno-miR-224-5p | 2.6831 | 0.001325876 | 0.067278458 | ** |
| rno-miR-3552 | 2.1934 | 0.005828178 | 0.123633421 | ** |
| rno-miR-146a-5p | 1.8849 | 0.004852554 | 0.123633421 | ** |
| rno-miR-143-3p | 1.8707 | 0.005146374 | 0.123633421 | ** |
| rno-miR-203a-3p | 1.8398 | 0.021972056 | 0.364927197 | * |
| rno-miR-152-3p | 1.7806 | 0.007926391 | 0.15527596 | ** |
| rno-miR-15b-3p | 1.7693 | 0.02359734 | 0.375590994 | * |
| rno-miR-350 | 1.6136 | 0.035334578 | 0.509351277 | * |
| rno-miR-155-5p | 1.5968 | 0.021653302 | 0.364927197 | * |
| rno-miR-204-5p | 1.5276 | 0.020695531 | 0.364927197 | * |
| rno-miR-211-5p | 1.5221 | 0.021462459 | 0.364927197 | * |
| rno-miR-143-5p | 1.4752 | 0.042010598 | 0.583565396 | * |
| rno-miR-34b-5p | 1.4535 | 0.028724738 | 0.447871427 | * |
| rno-miR-148a-5p | 1.39 | 0.048206921 | 0.646141888 | * |
| rno-miR-34c-5p | 1.3882 | 0.03491807 | 0.509351277 | * |
| rno-miR-199a-3p | 1.3272 | 0.043780362 | 0.597289222 | * |

| **miRNA_ID** | **log2(fold change)** | **P-value** | **FDR** | **Significance** |
| --- | --- | --- | --- | --- |
| **up-regulated MicroRNAs** | | | | |
| rno-miR-374-3p | -1.535 | 0.033082666 | 0.495591314 | * |
| rno-miR-539-3p | -1.6447 | 0.036071016 | 0.51033808 | * |
| rno-miR-335 | -1.9315 | 0.022886106 | 0.372020952 | * |
| rno-miR-219a-5p | -1.9649 | 0.030291249 | 0.46285028 | * |
| rno-miR-144-5p | -7.2192 | 0.004165659 | 0.113662984 | ** |
| rno-miR-3541 | -7.4594 | 0.001996045 | 0.084154325 | ** |
| rno-miR-3084b-5p | -8.0768 | 0.000164799 | 0.011446036 | ** |
| rno-miR-3084c-5p | -8.0768 | 0.000164799 | 0.011446036 | ** |
| rno-miR-3572 | -8.2143 | 7.45E-05 | 0.006320882 | ** |
