# Supplementary figures and images for: MicroRNA-204-5p reduction in rat hippocampus contributes to stress-induced pathology via targeting RGS12 signaling pathway
Source: J Neuroinflammation. 2021 Oct 21;18:243. doi: 10.1186/s12974-021-02299-5 (PMC8532383; doi:10.1186/s12974-021-02299-5)

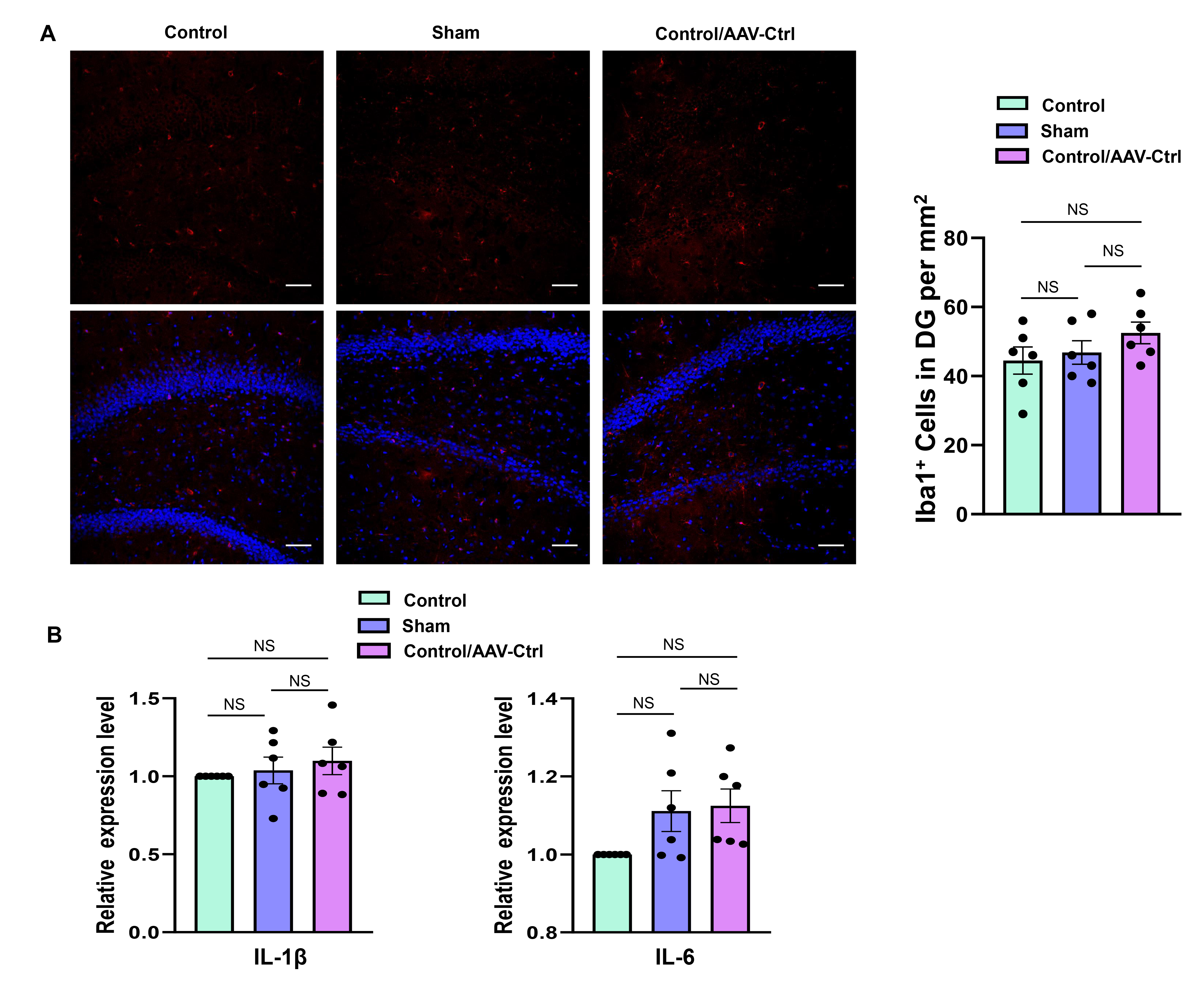

Supplement: Supplementary file 3 — Additional file 3: Fig. S1. Effects of sham operation and AAV injection on rats. (A) Immunofluorescent staining of Iba1 positive microglial cells within the DG region. Scale bar is 50 μm. (N = 6 per group). (B) Q-PCR analysis of IL-1βand IL-6 mRNA levels of each group. Band intensities were normalized to GAPDH (N = 6 per group). NS > 0.05. Data are presented as means ± SEMs. Student t-tests were employed for comparisons between the two groups. [file 12974_2021_2299_MOESM3_ESM.tif]

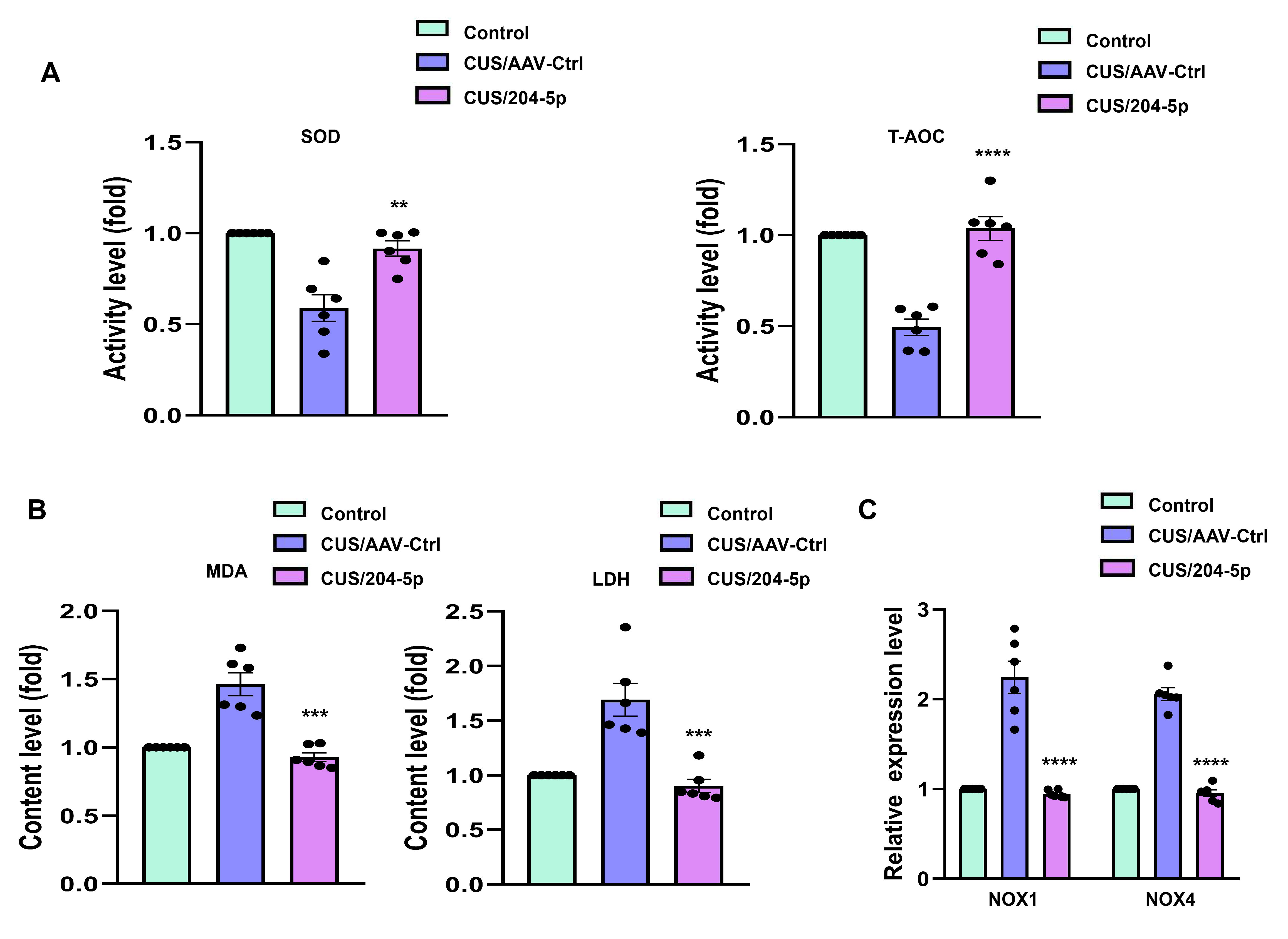

Supplement: Supplementary file 4 — Additional file 4: Fig. S2. MiR-204-5p overexpression within the hippocampal DG region alleviates oxidative stress in CUS rats. (A-B) Activity of antioxidant enzymes SOD and T-AOC. Contents of MDA and LDH were analyzed and levels were normalized to total protein content (N = 6 per group). (C) Q-PCR analysis of NOX1 and NOX4 mRNA levels of each group. Band intensities were normalized to GAPDH (N = 6 per group). **P < 0.01, ***P < 0.001, ****P < 0.0001, CUS + AAV-control vs CUS + AAV-miR-204-5p. Data are presented as means ± SEMs. Student t-tests were employed for comparisons between the two groups. [file 12974_2021_2299_MOESM4_ESM.tif]
