# Supplementary material for: MicroRNA-204-5p reduction in rat hippocampus contributes to stress-induced pathology via targeting RGS12 signaling pathway
Source: J Neuroinflammation. 2021 Oct 21;18:243. doi: 10.1186/s12974-021-02299-5 (PMC8532383; doi:10.1186/s12974-021-02299-5)
Supplement: Supplementary file 5 — Additional file 5. Figures S1, S2 legends. [file 12974_2021_2299_MOESM5_ESM.docx]

**MicroRNA-204-5p reduction in rat hippocampus contributes to stress-induced pathology via targeting RGS12 signaling pathway**

Tian Lan^a^, Ye Li ^a^, Cuiqin Fan^a^, Liyan Wang^b^, Wenjing Wang^a^, Shihong Chen ^c^﹡, Shu Yan Yu^a, d^﹡

a. Department of Physiology, Shandong University, School of Basic Medical Sciences, 44 Wenhuaxilu Road, Jinan, Shandong Province, 250012, PR China;

b. Morphological experimental center, Shandong University, School of Basic Medical Sciences, 44 Wenhuaxilu Road, Jinan, Shandong Province, 250012, PR China;

c. Department of Endocrinology, The Second Hospital, Cheeloo College of Medicine, Shandong University, 247 Beiyuan Street, Ji’nan, Shandong 250033, People’s Republic of China.

d. Shandong Provincial Key Laboratory of Mental Disorders, School of Basic Medical Sciences, 44 Wenhuaxilu Road, Jinan, Shandong Province, 250012, PR China;

﹡ Corresponding author: **Shu Yan Yu**,

E-mail address: shuyanyu@sdu.edu.cn

Tel: +86-0531-88383902; fax: +86-0531-88382502

**Shihong Chen,**

E-mail: chenshihong26@163.com

Tel: +86-0531- 88197777

**Fig. S1.** Effects of sham operation and AAV injection on rats. (A) Immunofluorescent staining of Iba1 positive microglial cells within the DG region. Scale bar is 50μm. (N=6 per group). (B) Q-PCR analysis of IL-1βand IL-6 mRNA levels of each group. Band intensities were normalized to GAPDH (N=6 per group). NS>0.05. Data are presented as means±SEMs. Student t-tests were employed for comparisons between the two groups.

**Fig. S2.** MiR-204-5p overexpression within the hippocampal DG region alleviates oxidative stress in CUS rats. (A-B) Activity of antioxidant enzymes SOD and T-AOC. Contents of MDA and LDH were analyzed and levels were normalized to total protein content (N= 6 per group). (C) Q-PCR analysis of NOX1 and NOX4 mRNA levels of each group. Band intensities were normalized to GAPDH (N=6 per group). **P < 0.01, ***P <0.001, ****P <0.0001, CUS + AAV-control vs CUS + AAV-miR-204-5p. Data are presented as means±SEMs. Student t-tests were employed for comparisons between the two groups.
